# Italy's health performance, 1990–2017: findings from the Global Burden of Disease Study 2017

**DOI:** 10.1016/S2468-2667(19)30189-6

**Published:** 2019-11-20

**Authors:** GBD 2017 Italy Collaborators, Lorenzo Monasta, Cristiana Abbafati, Giancarlo Logroscino, Giuseppe Remuzzi, Norberto Perico, Boris Bikbov, Giorgio Tamburlini, Ettore Beghi, Eugenio Traini, Sofia Boston Redford, Filippo Ariani, Antonio M Borzì, Cristina Bosetti, Giulia Carreras, Valeria Caso, Giulio Castelpietra, Massimo Cirillo, Sara Conti, Paolo Angelo Cortesi, Giovanni Damiani, Lucia Sara D'Angiolella, Jessica Fanzo, Carla Fornari, Silvano Gallus, Giorgia Giussani, Giuseppe Gorini, Giuseppe Grosso, Davide Guido, Carlo La Vecchia, Paolo Lauriola, Matilde Leonardi, Miriam Levi, Fabiana Madotto, Stefania Mondello, Luigi Naldi, Stefano Olgiati, Raffaele Palladino, Cristiano Piccinelli, Marco Piccininni, Elisabetta Pupillo, Alberto Raggi, Salvatore Rubino, Paola Santalucia, Marco Vacante, Simone Vidale, Francesco S Violante, Mohsen Naghavi, Luca Ronfani

## Abstract

**Background:**

Through a comprehensive analysis of Italy's estimates from the Global Burden of Diseases, Injuries, and Risk Factors Study (GBD) 2017, we aimed to understand the patterns of health loss and response of the health-care system, and offer evidence-based policy indications in light of the demographic transition and government health spending in the country.

**Methods:**

Estimates for Italy were extracted from GBD 2017. Data on Italy are presented for 1990 and 2017, on prevalence, causes of death, years of life lost, years lived with disability, disability-adjusted life-years (DALYs), life expectancy at birth and at age 65 years, healthy life expectancy, and Healthcare Access and Quality (HAQ) Index. We compared the estimates for Italy with those of 15 other western European countries.

**Findings:**

The quality of the universal health system and healthy behaviours contribute to favourable overall health, even in comparison with other western European countries. In 2017, life expectancy and HAQ Index score in Italy were among the highest globally, with life expectancy at birth reaching 85·3 years for females and 80·8 for males in 2017, ranking Italy eighth globally for females and sixth for males, and an HAQ Index score of 94·9 in 2016 compared with 81·54 in 1990, keeping Italy ranked as ninth globally. Between 1990 and 2017 age-standardised death rates for cardiovascular diseases decreased by 53·7% (95% uncertainty interval −56·1 to −51·4), for neoplasms decreased by 28·2% (−32·3 to −24·6), and for transport injuries decreased by 62·1% (−64·6 to −59·2). However, population ageing is causing an increase in the burden of specific diseases, such as Alzheimer's disease and other dementias (DALYs increased by 77·9% [68·4 to 87·2]) and pancreatic (DALYs increased by 39·7% [28·4 to 51·7]) and uterine cancers (DALYs increased by 164·7% [129·7 to 202·5]). Behavioural risk factors, which are potentially modifiable, still have a strong effect, particularly on cardiovascular diseases and neoplasms. For instance, in 2017, 44 400 (41 200 to 47 800) cancer deaths were attributed to smoking, 12 000 (9600 to 14 800) to alcohol use, and 9500 (5400 to 14 200) to high body-mass index, while 47 000 (31 100 to 65 700) deaths due to cardiovascular diseases could be attributed to high LDL cholesterol, 28 700 (19 700 to 38 500) to diets low in whole grains, and 15 900 (8500 to 24 900) to low physical activity.

**Interpretation:**

Italy provides an interesting example of the results that can be achieved by a mix of relatively healthy lifestyles and a universal health system. Two main issues require attention, population ageing and gradual decrease of public health financing, which both pose several challenges to the future of Italy's health status. Our findings should be useful to Italy's policy makers and health system experts elsewhere.

**Funding:**

Bill & Melinda Gates Foundation.

## Introduction

In 2015, according to the Organisation for Economic Co-operation and Development, life expectancy at birth in Italy was 82·7 years, the fourth highest globally and the second highest in the EU after Spain—approximately 2 years more than the average in the 28 countries that comprise the EU.^1^ In Italy, life expectancy at birth increased by 2·8 years between 2000 and 2015, and in 2015, 9·1% of the gross domestic product (GDP) went to health expenditures, a per capita expenditure below the EU average.^1^

Based on these data, Italy's overall health-care system performance appears quite satisfactory and was ranked second globally by WHO in 2010,^2^ first by the Bloomberg Global Health Index 2013,^3^ and ninth in the Global Burden of Diseases, Injuries, and Risk Factors Study (GBD) 2016 Healthcare Access and Quality (HAQ) Index globally.^4^

Since 1978, when the government established the *Servizio Sanitario Nazionale*—the Italian National Health Service (NHS)—Italy's health-care system has ensured universal coverage, free of charge at the point of service, for primary, secondary and tertiary care.^5^ Surgeries and treatment in hospital are provided for all citizens regardless of their income. Prescription drugs, specialist visits, and diagnostic tests are provided for free if prescribed by a physician, and require a copayment based on the medicine type and the patient's status—eg, people suffering from chronic diseases, older people (aged >65 years), people with a low income, pregnant women, and children are exempt from paying for drugs and laboratory examinations.^6^, ^7^

The system is strongly decentralised, with 19 regions and two autonomous provinces given large autonomy in managing health services, while the central government is still responsible for the overall system structure and guidelines on services that should be provided (ie, minimum assistance levels).^8^

**Research in context**

**Evidence before this study**

Italy has a universal health care system, good indicators for quality of life, and life expectancy in the country is ranked among the highest in the world, both for men and women. Economic data, however, tell us that government health expenditure is shrinking, the number of families in poverty is increasing, and the mean age of the population has increased substantially in the past 15 years. The aim of our study was to gain further insight into Italy's health performance.

**Added value of this study**

This is the first analysis of Italy using Global Burden of Diseases, Injuries, and Risk Factors Study (GBD) estimates. We used GBD 2017 estimates to do a detailed analysis of the main causes of disability and premature death and attributable risk factors. The estimates also provide an evidence base for analysis of crucial aspects of the health system, which to date has relied on a generally healthy population and now faces major challenges.

**Implications of all the available evidence**

Despite a general improvement of the population health status when considering age-standardised metrics, population ageing has a substantial bearing on the current burden of disease. Additionally, a considerable proportion of the burden of disease can still be attributed to behavioural risk factors, which could be addressed with awareness and prevention policies. The steady decrease in public health financing, accompanied by the increasing mean age of the population, poses new challenges that need to be addressed effectively.

Waiting times are usually up to a few months in public facilities, and up to a few weeks in publicly regulated private facilities. To shorten waiting time or gain access to a specific specialist, patients can opt for the private health care, provided by both public and private hospitals, which is paid for completely by the patient.^9^

Cost-containment measures have been progressively introduced by central and regional governments in Italy, with some acceleration over the past decade. In response to national targets to transform part of acute care into long-term care facilities for chronic conditions, the number of hospital beds has decreased substantially, from 4·7 per 1000 people in 2000, to 3·0 per 1000 people in 2016.^10^

A spending review in 2012 led to decreased funding for the NHS, with respect to the requirements established by the State-Regions Conference, of €0·9 billion in 2012, €1·8 billion in 2013, and €2·0 billion in 2014, representing reductions of the required funding of 0·8% in 2012, 1·6% in 2013, and 1·8% in 2014.^11^ As a consequence of the economic crisis, which caused a decrease in GDP of over 8% between 2008 and 2015, the public financing of the health systems was further reduced.^12^

The 2018 Economic and Financial Document set public health spending as 6·4% of GDP in 2019 and 6·3% of GDP in 2020.^13^ In 2015, for the first time in 15 years, a reduction was seen in life expectancy in Italy, with female life expectancy decreasing from 85·6 years in 2014 to 84·9 years, and male life expectancy decreasing from 80·7 years in 2014 to 80·3 years;^14^ such reductions were fortunately limited to 2015.

To understand the patterns of disease burden and examine the response of the health-care system to this burden, we provide a thorough description and analysis of Italy's GBD 2017 estimates, including temporal trends and comparisons with other countries for the main GBD measures.

## Methods

### Overview

We extracted estimates on Italy and other European countries from GBD 2017. Methods for the generation of GBD 2017 estimates are described in detail elsewhere^15^, ^16^, ^17^, ^18^, ^19^ and are compliant with the Guidelines for Accurate and Transparent Health Estimates Reporting.^20^ GBD 2017 comprehensively and systematically analysed 282 causes of death, 359 diseases and injuries, and 84 behavioural, metabolic, and environmental and occupational risks for 195 countries and territories, including Italy and all European countries.

Mortality-related models (all-cause mortality, cause-specific mortality, and years of life lost [YLLs]) were informed by vital registration, sample registration, surveys, surveillance, registries, and verbal autopsy data. Estimates were generated using standardised approaches of data identification, extraction, and processing to address issues of incompleteness, variation in classification systems, and coding practices.^15^, ^16^, ^17^, ^18^, ^19^

### GBD measures

We present data on Italy for 1990 and 2017, including prevalence data, causes of death, YLLs, years lived with disability (YLDs), disability-adjusted life-years (DALYs), life expectancy at birth and at 60 years old, and healthy life expectancy (HALE).^15^, ^16^, ^17^, ^18^, ^19^ Briefly, YLLs are years of life lost due to premature death, calculated as the difference between the corresponding standard life expectancy for that person's age and sex, and the age of actual death.^15^

YLDs, described in detail in the GBD 2017 incidence and prevalence capstone paper,^16^ are years lived with disability (in which the disability equates to a fraction of a year lived in full health) and are the product of the mean duration of the condition in years and the disability weight of that condition.

DALYs are the sum of YLLs and YLDs, and represent the loss in years due to premature death and the fraction of years lived in less than full health.

The HAQ Index is a GBD measure which relies on GBD 2016 estimates, and has not yet been issued for 2017.^4^ It is based on mortality-to-incidence ratios from causes that, in the presence of quality health care, should not result in death.

We also present estimates of infant and under-5 mortality, which are key epidemiological indicators of wellbeing.

In this analysis, data on health financing, including government and out-of-pocket spending, was taken from the GBD analysis on financing global health, 2016, presenting estimates up to 2015.^21^

### Data extraction and presentation

Data visualisation tools, from which most of the data were extracted, are available online and provide data from 1990 to 2017. We extracted Italy's financial data from the Financing Global Health project.^21^ We present results for the time period between 1990 and 2017. We present count estimates, all-age rates per 100 000 population, age-standardised rates per 100 000 population, and percentage changes from 1990 to 2017 for YLDs, YLLs, DALYs, and deaths.

### GBD causes hierarchy

In GBD 2017, diseases and injuries and causes of death, were aggregated in three Level 1 causes (communicable, maternal, neonatal, and nutritional conditions; non-communicable diseases; and injuries), 22 Level 2 causes, 169 Level 3 causes, and 293 Level 4 causes.^15^, ^16^, ^17^, ^19^ For example, migraine is a Level 4 cause in the Level 3 headache disorders group in the Level 2 neurological disorders group, and in the Level 1 non-communicable diseases group. Only the top 20 causes of YLDs, YLLs, DALYs, and deaths are presented in our analysis.

In the Results section, we used the Level 3 cause categorisation, unless otherwise specified. The 84 risk factors are aggregated in three Level 1 groups (metabolic, environmental and occupational, and behavioural risks), 19 Level 2 groups, 50 Level 3 groups, and 67 Level 4 groups.^18^ We used Level 2 aggregation of risk factors in the comparison among selected European countries, and the top 20 Level 3 risk factors for other anlyses.

### Comparisons with European countries

The European countries we selected for comparison with Italy were Austria, Belgium, Denmark, Finland, France, Germany, Greece, Ireland, Luxembourg, the Netherlands, Portugal, Spain, Sweden, and the UK. These countries form the EU15 countries together with Italy and we chose them as our comparator countries because they are founding members of the EU or have joined the EU (no later than 1995). We compared the rankings of each country in 2017 by age-standardised YLDs and DALYs, by risk factors attributed to DALYs, and by age-standardised rates of death, YLLs, YLDs, and DALYs both in 1990 and 2017.

### Socio-demographic Index

We present some estimates for Italy in comparison with expected values corresponding to the level of the Socio-demographic Index (SDI), a composite indicator of development status built as geometric mean of 0 to 1 indices of total fertility rate in women younger than 25 years, mean education for the population aged 15 years and older, and lag-distributed income per capita.

### Uncertainty analysis

All data are presented with 95% uncertainty intervals (UIs),^15^, ^16^, ^17^, ^18^, ^19^ some of which were specifically extracted for the scope of this Article by the Institute for Health Metrics and Evaluation (IHME). Differences in median percentage changes were considered significant if 95% UIs did not overlap.

### Role of the funding source

The funder of the study had no role in study design, data collection, analysis and interpretation, or writing of the report. The corresponding author had full access to all the data in the study and had final responsibility to submit for publication.

## Results

In terms of SDI, Italy is ranked 28th globally according to GBD 2017, with an SDI value of 0·8434; ranking lower than Poland (ranked 27th; 0·8438) and higher than the UK (ranked 29th; 0·8431). Life expectancy at birth reached 85·3 years for females and 80·8 for males in 2017, which ranks Italy eighth globally for females and sixth for males. Life expectancy at birth in Italy is 4 years higher than expected on the basis of its SDI value (these estimates are available on the GBD Compare website).

Italy's total fertility rate (ie, number of children born or likely to be born to a woman in her life time) has remained stable since 1990 and was 1·3 in 2017, which was the tenth lowest total fertility rate globally. The country's population remained relatively stable from 1990 to 2017, with a population increase of just 3·8 million (56·8 million [95% UI 52·8–60·7] in 1990; 60·6 million [60·1–61·0] in 2017). These factors have led to a rapidly changing population structure, with the mean age increasing from 38·4 years (35·7–41·0) in 1990 to 44·4 years (44·1–44·7) in 2017.

In 2017, under-5 and infant mortality in Italy were lower than expected on the basis of SDI level (under-5 mortality: 3·2 deaths per 1 000 livebirths; infant mortality: 2·7 deaths per 1000 livebirths). Finally, with an HAQ Index of 94·9 in 2016, Italy had the ninth highest HAQ Index score globally, exceeding the score expected on the basis of its SDI value by 0·6.^4^

With an HAQ Index of 81·54, Italy also ranked ninth in 1990, showing a constant increase from 1990 to 2016 in line with the other top 12 countries globally (appendix p 1). In Italy in 2015, the overall per capita expenditure for health was divided such that 75% was from government health spending, 23% was from out-of-pocket spending, and 2% was from prepaid private spending. From 2010 to 2015, government health spending as percentage of GDP decreased from 7·0% to 6·7%, while out-of-pocket spending increased from 1·8% to 2·0%.

The overall number of YLLs decreased by 20·1% from 1990 to 2017 (table 1), while the total number of deaths increased by 14·9% (appendix p 9), indicating that people are dying at older ages, closer to their potential life expectancy. The overall age-standardised death rate decreased by 41·3% between 1990 and 2017, while age-standardised YLL rates decreased by 49·5% in the same period.

**Table 1:** Number of YLLs by Level 3 causes in 1990 and 2017, and YLL rates and age-standardised YLL rates for 2017 with percentage change between 1990 and 2017, ordered by ranking of top 20 causes of YLLs in 2017

	**Rank of cause of YLLs**		**YLLs (in thousands)**		**All-age YLL rate per 100 000 people**		**Age-standardised YLL rate per 100 000 people**	
	1990	2017	2017	Percentage change 1990–2017	2017	Percentage change 1990–2017	2017	Percentage change 1990–2017
All causes	..	..	8005 (7615 to 8403)	−20·1% (−23·9 to −16·3)	13 209 (12 566 to 13 866)	−25·1% (−28·6 to −21·5)	6709 (6384 to 7038)	−49·5% (−52·0 to −47·1)
Ischaemic heart disease	1 (1 to 1)	1 (1 to 1)	1028 (957 to 1118)	−35·3% (−39·5 to −30·4)	1697 (1579 to 1845)	−39·3% (−43·3 to −34·8)	690 (640 to 743)	−60·9% (−63·6 to −58·0)
Tracheal, bronchus, and lung cancer	3 (3 to 3)	2 (2 to 2)	602 (564 to 645)	−15·5% (−21·3 to −9·4)	994 (931 to 1064)	−20·8% (−26·2 to −15·0)	479 (449 to 512)	−41·2% (−45·2 to −37·1)
Alzheimer's disease and other dementias	6 (6 to 6)	3 (3 to 4)	581 (542 to 619)	75·6% (64·2 to 86·1)	959 (895 to 1022)	64·6% (53·9 to 74·5)	302 (281 to 323)	−15·0% (−20·7 to −9·6)
Stroke	2 (2 to 2)	4 (3 to 4)	580 (539 to 640)	−37·7% (−42·1 to −32·6)	956 (889 to 1056)	−41·6% (−45·7 to −36·8)	369 (343 to 403)	−64·1% (−66·7 to −61·3)
Colon and rectum cancer	8 (8 to 8)	5 (5 to 5)	319 (295 to 345)	6·2% (−1·9 to 14·8)	526 (486 to 569)	−0·4% (−8·1 to 7·6)	245 (226 to 265)	−28·1% (−33·8 to −22·1)
Chronic obstructive pulmonary disease	11 (11 to 13)	6 (6 to 6)	252 (233 to 274)	−4·7% (−12·6 to 5·1)	416 (385 to 452)	−10·6% (−18·1 to −1·5)	153 (141 to 167)	−45·5% (−50·3 to −39·4)
Breast cancer	9 (9 to 9)	7 (7 to 7)	240 (217 to 262)	−12·9% (−21·6 to −4·8)	396 (358 to 433)	−18·4% (−26·5 to −10·8)	213 (192 to 233)	−38·2% (−44·3 to −32·4)
Diabetes	10 (10 to 11)	8 (8 to 8)	226 (210 to 243)	−16·1% (−22·8 to −9·4)	372 (346 to 401)	−21·4% (−27·6 to −15·1)	155 (144 to 167)	−46·9% (−51·1 to −42·5)
Cirrhosis and other chronic liver diseases	5 (5 to 5)	9 (9 to 10)	214 (193 to 234)	−47·8% (−52·8 to −42·8)	354 (318 to 387)	−51·1% (−55·7 to −46·4)	185 (166 to 203)	−63·2% (−67·0 to −59·4)
Pancreatic cancer	16 (16 to 17)	10 (9 to 10)	208 (194 to 226)	39·3% (28·0 to 51·4)	344 (320 to 374)	30·6% (20·0 to 41·9)	163 (151 to 177)	−3·8% (−11·3 to 4·5)
Stomach cancer	7 (7 to 7)	11 (11 to 12)	185 (172 to 200)	−42·9% (−47·2 to −38·5)	306 (284 to 329)	−46·5% (−50·5 to −42·4)	144 (133 to 155)	−60·8% (−63·9 to −57·7)
Hypertensive heart disease	19 (19 to 25)	12 (11 to 20)	184 (86 to 211)	44·3% (−17·9 to 64·1)	304 (141 to 349)	35·3% (−23·1 to 53·9)	105 (58 to 119)	−22·9% (−50·8 to −13·3)
Liver cancer	15 (15 to 15)	13 (12 to 13)	179 (162 to 199)	−1·2% (−10·8 to 9·3)	296 (267 to 328)	−7·4% (−16·4 to 2·4)	141 (127 to 157)	−30·9% (−37·7 to −23·5)
Road injuries	4 (4 to 4)	14 (13 to 15)	159 (147 to 170)	−66·3% (−68·5 to −63·6)	262 (243 to 281)	−68·4% (−70·5 to −65·9)	264 (246 to 284)	−67·1% (−69·5 to −64·5)
Lower respiratory infections	22 (20 to 22)	15 (15 to 16)	140 (129 to 152)	21·4% (9·9 to 33·3)	231 (213 to 251)	13·8% (3·0 to 25·0)	108 (100 to 117)	−41·8% (−48·5 to −35·0)
Chronic kidney disease	20 (18 to 20)	16 (14 to 17)	139 (129 to 150)	12·3% (4·1 to 22·0)	230 (214 to 247)	5·3% (−2·4 to 14·3)	89 (83 to 96)	−37·4% (−42·0 to −32·1)
Self-harm	14 (14 to 14)	17 (16 to 18)	137 (126 to 150)	−27·1% (−33·4 to −19·9)	226 (208 to 247)	−31·6% (−37·6 to −24·9)	192 (177 to 210)	−32·3% (−38·3 to −25·7)
Leukaemia	18 (17 to 18)	18 (17 to 19)	132 (122 to 142)	−9·6% (−16·7 to −2·3)	217 (201 to 235)	−15·3% (−22·0 to −8·4)	137 (126 to 149)	−36·2% (−41·7 to −30·2)
Prostate cancer	27 (23 to 28)	19 (14 to 18)	114 (97 to 179)	23·7% (7·0 to 62·7)	189 (161 to 295)	16·0% (0·3 to 52·5)	72 (61 to 116)	−24·2% (−35·0 to 1·2)
Other malignant neoplasms	21 (21 to 24)	20 (21 to 22)	100 (83 to 113)	−14·4% (−24·4 to 10·5)	165 (137 to 186)	−19·8% (−29·2 to 3·5)	107 (89 to 124)	−34·4% (−43·4 to −13·3)

Data in parentheses are 95% uncertainty intervals. YLLs=years of life lost.

Ischaemic heart disease, Alzheimer's disease and other dementias, and stroke are the first, second, and third Level 3 causes of death and the first, third, and fourth Level 3 causes of YLLs in 2017 (table 1; appendix p 9). Among Level 2 causes, neoplasms are the first leading cause of YLLs and deaths, and cardiovascular diseases are the second leading cause of YLLs and deaths. Despite the overall decrease between 1990 and 2017 in both the number of YLLs and age-standardised YLL rate, in 2017 cardiovascular diseases, which comprise 32% of all YLLs in 1990, still accounted for 28% of YLLs (full data for YLLs are available online). Also in terms of age-standardised death rates, a decrease was seen for both neoplams (−28·2%, 95% UI −32·3 to −24·6) and cardiovascular diseases (−53·7%, −56·1 to −51·4) between 1990 and 2017.

The second leading cause of Level 3 YLLs in 2017 was tracheal, bronchus, and lung cancer (table 1). Taken together, among Level 2 causes, neoplasms were the top cause of YLLs in 2017, comprising 38% of all YLLs. The number of YLLs caused by neoplasms decreased by 5·9% (95% UI 0·9–11·2) between 1990 and 2017. However, significant increases in number of YLLs were registered for pancreatic, prostate, kidney, cervical, uterine, and skin cancers (data not shown; full YLLs Level 2 data are available online).

At the same time, the age-standardised YLL rate decreased between 1990 and 2017 for all neoplasms except uterine cancer (for all neoplasms −34·3% [95% UI −38·2 to −30·8], for uterine cancer 68·6% [47·6% to 92·6]). Age-standardised death rates also showed significant increases only for uterine cancer (71% increase; from 0·6 deaths [0·6–0·7] per 100 000 in 1990 to 1·1 deaths [1·0–1·2] per 100 000 in 2017). For number of deaths, pancreatic cancer had an increase of 65·6% (53·1 to 79·1) reaching almost 13 000 deaths in 2017 (appendix p 9), whereas uterine cancer, even though the number of deaths was lower than for pancreatic cancer (1600 in 2017), the number of deaths had increased by 177% (146 to 213) since 1990.

Alzheimer's disease and other dementias became the third leading Level 3 cause of YLLs in 2017, increasing from sixth in 1990, and accounted for 7% of total YLLs (table 1). The number of YLLs due to this cause increased by 75·6% between 1990 and 2017, while the number of deaths increased by 117·7% (95% UI 104·7 to 129·5) and death rates increased by 104·0% (91·9 to 115·2) during this period (table 1; appendix p 9). However, age-standardised rates attributable to this cause decreased significantly both for YLLs (−15·0%, −20·7 to −9·6) and deaths (−12·6%, −17·9 to −7·6).

Overall, both the number of deaths and YLLs caused by road injuries decreased significantly between 1990 and 2017 (number of deaths in 1990: 11 243 [11 009 to 11 474]; in 2017: 5710 [95% UI 5333 to 6090]; percentage change in number of deaths −49·2% [–52·5 to −45·6]), with a decrease in the age-standardised death rate of 62·1% (−64·6 to −59·2) and shifted in rank from 12th to 23rd as a cause of death. YLLs decreased by 66·3% [–68·5 to −63·6]), from 470 389 (460 429 to 480 378) in 1990 to 158 685 (147 495 to 170 172) in 2017, shifting from fourth to 14th as a cause of YLLs.

The ranking of diabetes for number of YLLs did not vary substantially (tenth in 1990 to eighth in 2017), with a decrease of 16·1% (−22·8 to −9·4) in absolute numbers and a decrease of 46·9% (−51·1 to −42·5) in age-standardised YLL rate (table 1). The number of deaths caused by diabetes increased by 12·2% (3·2 to 21·3) between 1990 and 2017, even though the age-standardised death rate decreased by 40·5% (−45·1 to −35·8; appendix p 9).

The rank of chronic kidney disease by number of YLLs increased from 20th to 16th and by deaths increased from 15th to ninth between 1990 and 2017 (table 1; appendix p 9). During this period, the total number of deaths from chronic kidney disease increased by 74·2% (95% UI 61·1 to 89·2), while age-standardised death rates decreased by 19·5% (−25·3 to −12·9; from 9·1 [8·8–9·4] per 100 000 population to 7·3 [6·8–7·9] per 100 000 population). Drug use disorders had a decrease in the number of deaths of 35·4% (−43·4 to −26·9), with the number of YLLs decreasing even more (−62·5% [–68·0 to −55·9]), but still being responsible for 147 000 DALYs (111 000 to 184 000).

Finally, the ranking of neonatal disorders (including preterm births, encephalopathies due to birth asphyxia and trauma, sepsis and other infections, haemolytic diseases and other neonatal jaundice, and other neonatal disorders) decreased significantly in terms of the number of YLLs, from 13th with 250 959 YLLs in 1990, to 32nd with 63 501 YLLs in 2017 (−74·7% [–67·3 to −80·8]).

Even in terms of number of deaths, a considerable reduction was seen from 2857 in 1990 to 723 in 2017. This reduction was coupled with a significant reduction in YLLs caused by congenital defects, reflected in a decrease of 63·9% (95% UI −50·5 to −70·0) in the number of YLLs, and a decrease of 55·5% (−42·7 to −62·8) in the number of deaths. These results are only partially explained by a reduction in the number of livebirths, which decreased from 569 255 in 1990 to 458 151 in 2017, an almost 20% decrease (full data are available on the Istat website).

For YLDs, the ranking of the most prevalent diseases and injuries (not discussed here) is shown in the appendix (p 10), and the leading causes of YLDs for 2017 and a comparison with 1990 is shown in table 2;. Between 1990 and 2017, the number of YLDs increased by 17·6% (95% UI 15·7 to 19·5) but the age-standardised YLD rate decreased by 2·8% (−4·4 to −1·1). Low back pain was the leading cause of YLDs for 2017. Age-standardised YLD rates for low back pain decreased significantly between 1990 and 2017 (−6·2%, −11·1 to −1·1), although the number of YLDs increased significantly (11·2%, 5·0 to 17·3).

**Table 2:** Number of YLDs, YLD rates and age-standardised YLD rates in 2017 with percentage change between 1990 and 2017, ordered by ranking of top 20 Level 3 causes of YLDs in 2017

	**Rank of cause of YLDs**		**YLDs (in thousands)**		**YLD rate per 100 000 people**		**Age-standardised YLD rate per 100 000 people**	
	1990	2017	2017	Percentage change, 1990–2017	2017	Percentage change, 1990–2017	2017	Percentage change, 1990–2017
All causes	..	..	8488 (6369 to 10 878)	17·6% (15·7 to 19·5)	14 008 (10 510 to 17 951)	10·2% (8·5 to 12·0)	10 313 (7684 to 13 277)	−2·8% (−4·4 to −1·1)
Low back pain	1 (1 to 1)	1 (1 to 1)	1057 (758 to 1421)	11·2% (5·0 to 17·3)	1744 (1251 to 2345)	4·2% (−1·5 to 9·9)	1284 (917 to 1733)	−6·2% (−11·1 to −1·1)
Headache disorders	2 (2 to 2)	2 (2 to 2)	723 (477 to 1035)	4·5% (1·1 to 8·2)	1194 (787 to 1708)	−2·0% (−5·2 to 1·4)	1110 (730 to 1598)	0·6% (−2·5 to 3·9)
Diabetes	8 (8 to 8)	3 (3 to 3)	507 (343 to 697)	89·4% (69·6 to 116·1)	838 (566 to 1151)	77·5% (59·0 to 102·5)	446 (299·9 to 623)	38·2% (24·5 to 55·8)
Neck pain	4 (4 to 4)	4 (4 to 4)	443 (309 to 620)	25·3% (21·9 to 28·9)	731 (510 to 1024)	17·4% (14·3 to 20·8)	481 (336 to 667)	−0·1% (−1·7 to 1·9)
Age-related and other hearing loss	6 (6 to 7)	5 (5 to 5)	427 (300 to 588)	54·8% (50·3 to 60·2)	705 (495 to 971)	45·1% (40·9 to 50·2)	328 (225 to 460)	0·2% (−2·0 to 2·3)
Depressive disorders	3 (3 to 3)	6 (6 to 6)	383 (270 to 519)	−1·7% (−6·7 to 3·3)	632 (446 to 856)	−7·9% (−12·6 to −3·2)	528 (372 to 726)	−10·8% (−15·0 to −6·2)
Falls	7 (6 to 7)	7 (7 to 7)	334 (237 to 456)	23·7% (20·1 to 27·9)	551 (392 to 752)	15·9% (12·6 to 19·9)	354 (249 to 487)	−7·8% (−9·9 to −5·5)
Anxiety disorders	5 (5 to 5)	8 (8 to 8)	319 (224 to 423)	2·0% (−3·4 to 7·9)	526 (370 to 699)	−4·4% (−9·4 to 1·2)	499 (351 to 667)	−2·6% (−8·0 to 3·2)
Other musculoskeletal disorders	9 (9 to 9)	9 (9 to 9)	251 (164 to 362)	26·7% (14·3 to 40·9)	414 (270 to 598)	18·7% (7·2 to 32·1)	313 (208 to 448)	9·8% (0·7 to 20·7)
Blindness and vision impairment	11 (10 to 11)	10 (11 to 11)	215 (149 to 294)	28·0% (24·3 to 32·2)	354 (245 to 486)	20·0% (16·5 to 23·9)	200 (135 to 282)	−10·6% (−13·3 to −7·9)
Chronic obstructive pulmonary disease	13 (12 to 19)	11 (10 to 14)	187 (155 to 221)	46·9% (30·1 to 63·7)	308 (256 to 365)	37·7% (21·9 to 53·4)	151 (124 to 180)	0·9% (−10·3 to 11·8)
Alzheimer's disease and other dementias	22 (19 to 22)	12 (12 to 13)	178 (129 to 231)	85·7% (70·5 to 102·9)	294 (213 to 381)	74·1% (59·8 to 90·2)	93 (67 to 121)	−9·5% (−16·4 to −1·7)
Oral disorders	10 (10 to 11)	13 (12 to 13)	172 (107 to 261)	−2·3% (−4·5 to 0·0)	285 (176 to 430)	−8·4% (−10·5 to −6·3)	182 (109·7 to 280)	−21·2% (−24·0 to −18·9)
Osteoarthritis	20 (12 to 29)	14 (10 to 20)	148 (75 to 295)	51·8% (45·0 to 58·4)	245 (123 to 487)	42·3% (35·9 to 48·5)	116 (58 to 231)	4·0% (−0·5 to 8·5)
Drug use disorders	12 (13 to 13)	15 (14 to 17)	124 (89 to 162)	−11·0% (−18·6 to −2·1)	205 (147 to 268)	−16·5% (−23·7 to −8·3)	223 (158 to 291)	−4·7% (−13·5 to 5·5)
Stroke	15 (16 to 20)	16 (15 to 22)	121 (87 to 154)	5·8% (−3·7 to 15·0)	200 (144 to 254)	−0·8% (−9·7 to 7·8)	89 (65 to 114)	−31·0% (−36·3 to −25·7)
Bipolar disorder	16 (14 to 17)	17 (16 to 21)	117 (73 to 173)	2·4% (−4·9 to 10·3)	193 (120 to 285)	−4·0% (−10·8 to 3·3)	179 (110 to 268)	−0·7% (−8·0 to 7·8)
Road injuries	14 (15 to 15)	18 (17 to 19)	116 (83 to 157)	−2·6% (−4·5 to −0·6)	192 (137 to 259)	−8·7% (−10·5 to −6·8)	131 (93 to 177)	−21·3% (−22·9 to −19·6)
Neonatal disorders	17 (14 to 18)	19 (16 to 20)	115 (83 to 157)	2·3% (−9·8 to 15·7)	190 (137 to 259)	−4·1% (−15·4 to 8·5)	226 (162 to 308)	3·1% (−9·4 to 17·0)
Upper digestive system diseases	18 (16 to 22)	20 (15 to 23)	114 (68 to 180)	12·2% (6·9 to 16·6)	188 (112 to 297)	5·1% (0·2 to 9·3)	134 (80 to 215)	−5·8% (−9·8 to −2·5)

Data in parentheses are 95% uncertainty intervals. YLDs= years lived with disability.

In the 15–49 years age group, low back pain and headache disorders were the leading causes of YLDs in the in both 1990 and 2017, with non-significant changes in YLD rates since 1990 (low back pain, percentage change: −0·10% [–0·90 to 0·07]; headache disorders, percentage change: 0·04% [–0·01 to 0·08]; full data are available online).

In 2017, diabetes was the third leading cause in number of YLDs, with an increase of 89·4% (95% UI 69·6 to 116·1) since 1990 in terms of absolute numbers and 38·2% (24·5 to 55·8) in terms of age-standardised rates (table 2). Alzheimer's disease and other dementias ranked 12th in terms of number of YLDs, with an 85·7% (70·5 to 102·9) increase in the number of YLDs between 1990 and 2017, even while the age-standardised YLD rate decreased by 9·5% (−16·4 to −1·7).

**Figure 1:**
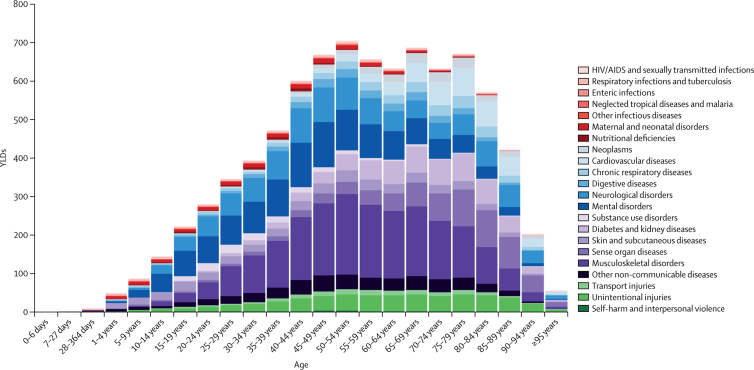
Number of YLDs for 22 Level 2 diseases and injuries in Italy in 2017, for both sexes combined, by age group YLDs=years lived with disability.

In 2017, if considered together (ie, at Level 2), musculoskeletal disorders were the most important cause of YLDs, followed by mental disorders and neurological disorders (figure 1). The prevalence of cardiovascular diseases, skin and subcutaneous diseases, and sense organ diseases appear to increase with age, whereas nutritional deficiencies, enteric infections, other infectious diseases, and other non-communicable diseases occur more often in younger age groups. YLDs due to musculoskeletal disorders and mental disorders increase with age up to 50–54 years, and then appear to gradually decrease. Number of YLDs due to neoplasms increased by 68·2% (95% UI 82·5–55·9).

For DALYs, in 1990 the YLD component made up 42% of all DALYs, reaching 51% in 2017, which is primarily due to a 20·1% decrease in YLLs between 1990 and 2017 and a 17·6% increase in YLDs over the same period. As a result, the disease and injuries that contribute the most to YLLS and YLDs contribute substantially to national DALYs.

**Figure 2:**
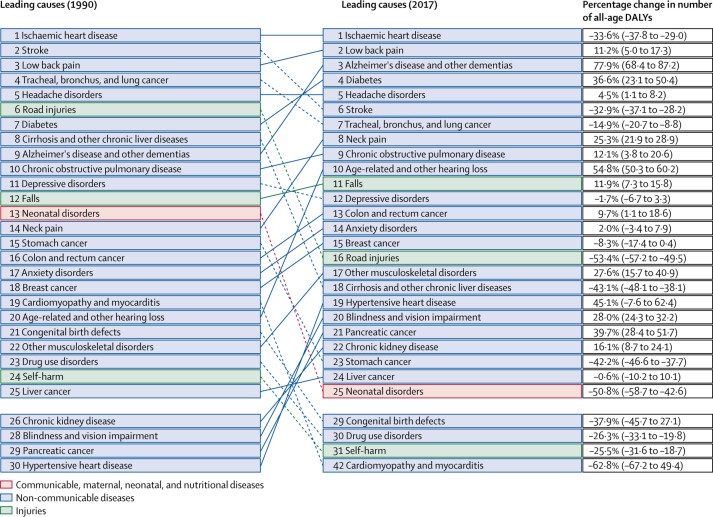
Top 25 causes of DALYs in Italy, in 1990–2017, and percentage change between 1990 and 2017 Dotted lines show a decrease in rank and solid lines show an increase in rank. DALYs=disability-adjusted life-years.

In 2017, ischaemic heart disease, as the the leading cause of YLLs, was the top contributor to premature death and the leading cause of DALYs, followed by low back pain, the top contributor to non-fatal disability. Alzheimer's disease and other dementias was third highest contributor and diabetes was fourth highest contributor to DALYs (figure 2, table 3).

**Table 3:** DALYs, DALY rates, and age-standardised DALY rates in 2017, with percentage change between 1990 and 2017, ordered by ranking in top 20 Level 3 causes of DALYs in 2017

	**Rank by number of DALYs in 2017**		**DALYs (in thousands)**		**All-age DALY rate per 100 000 people**		**Age-standardised DALY rate per 100 000 people**	
	1990	2017	2017	Percentage change, 1990–2017	2017	Percentage change, 1990–2017	2017	Percentage change, 1990–2017
All causes	..	..	16 493 (14 334 to 18 969)	−4·3% (−7·9 to −0·9)	27 217 (23 654 to 31 303)	−10·3% (−13·6 to −7·1)	17 022 (14 382 to 20 024)	−28·8% (−32·5 to −25·3)
Ischaemic heart disease	1 (1 to 1)	1 (1 to 2)	1110 (1034 to 1204)	−33·6% (−37·8 to −29·0)	1831 (1706 to 1987)	−37·8% (−41·7 to −33·4)	749 (697 to 805)	−59·7% (−62·3 to −56·9)
Low back pain	3 (2 to 4)	2 (1 to 2)	1057 (758 to 1421)	11·2% (5·0 to 17·3)	1744 (1251 to 2345)	4·2% (−1·5 to 9·9)	1284 (917 to 1733)	−6·2% (−11 to −1·1)
Alzheimer's disease and other dementias	9 (9 to 11)	3 (3 to 5)	759 (695 to 825)	77·9% (68·4 to 87·2)	1253 (1148 to 1362)	66·7% (57·9 to 75·4)	396 (362 to 429)	−13·8% (−18·4 to −9·1)
Diabetes	7 (6 to 7)	4 (4 to 6)	733 (570 to 921)	36·6% (23·1 to 50·4)	1210 (941 to 1520)	28·0% (15·4 to 41·0)	601 (457 to 777)	−2·2% (−12·8 to 8·1)
Headache disorders	5 (4 to 6)	5 (3 to 7)	723 (477 to 1035)	4·5% (1·1 to 8·2)	1194 (787 to 1708)	−2·0% (−5·2 to 1·4)	1110 (730 to 1598)	0·6% (−2·5 to 3·9)
Stroke	2 (2 to 3)	6 (4 to 6)	700 (647 to 764)	−32·9% (−37·1 to −28·2)	1156 (1068 to 1261)	−37·1% (−41·0 to −32·7)	458 (422 to 498)	−60·4% (−62·9 to −57·8)
Tracheal, bronchus, and lung cancer	4 (3 to 5)	7 (5 to 7)	612 (573 to 654)	−14·9% (−20·7 to −8·8)	1011 (946 to 1079)	−20·2% (−25·7 to −14·6)	487 (456 to 520)	−40·9% (−44·9 to −36·6)
Neck pain	14 (9 to 17)	8 (8 to 11)	443 (309 to 620)	25·3% (21·9 to 28·9)	731 (510 to 1024)	17·4% (14·3 to 20·8)	481 (336 to 667)	−0·1% (−1·7 to 1·9)
Chronic obstructive pulmonary disease	10 (10 to 13)	9 (8 to 12)	438 (399 to 477)	12·1% (3·8 to 20·6)	723 (658 to 788)	5·0% (−2·7 to 13·1)	304 (273 to 335)	−29·4% (−35 to −23·7)
Age-related and other hearing loss	20 (16 to 21)	10 (9 to 12)	427 (300 to 588)	54·8% (50·3 to 60·2)	705 (495 to 971)	45·1% (40·9 to 50·2)	328 (225 to 460)	0·2% (−2·0 to 2·3)
Falls	12 (10 to 14)	11 (9 to 10)	418 (321 to 542)	11·9% (7·3 to 15·8)	690 (529 to 894)	4·9% (0·6 to 8·6)	422 (318 to 554)	−19·2% (−23·1 to −16·1)
Depressive disorders	11 (8 to 16)	12 (11 to 13)	383 (270 to 519)	−1·7% (−6·7 to 3·3)	632 (446 to 856)	−7·9% (−12·6 to −3·2)	528 (372 to 726)	−10·8% (−15·0 to −6·2)
Colon and rectum cancer	16 (13 to 18)	13 (10 to 15)	345 (319 to 376)	9·7% (1·1 to 18·6)	570 (526 to 621)	2·8% (−5·2 to 11·2)	265 (244 to 289)	−25·9% (−31·7 to −19·6)
Anxiety disorders	17 (14 to 18)	14 (13 to 16)	319 (224 to 423)	2·0% (−3·4 to 7·9)	526 (370 to 699)	−4·4% (−9·4 to 1·2)	499 (351 to 667)	−2·6% (−8·0 to 3·2)
Breast cancer	18 (15 to 19)	15 (14 to 17)	276 (248 to 304)	−8·3% (−17·4 to 0·4)	456 (410 to 503)	−14·0% (−22·6 to −5·9)	245 (219 to 271)	−34·9% (−41·4 to −28·4)
Road injuries	6 (5 to 7)	16 (15 to 16)	275 (241 to 316)	−53·4% (−57·2 to −49·5)	454 (398 to 522)	−56·3% (−59·8 to −52·7)	396 (354 to 447)	−59·3% (−62·1 to −56·2)
Other musculoskeletal disorders	22 (21 to 28)	17 (14 to 20)	266 (180 to 378)	27·6% (15·7 to 40·9)	440 (297 to 624)	19·6% (8·5 to 32·0)	327 (223 to 463)	9·3% (0·6 to 19·1)
Cirrhosis and other chronic liver diseases	8 (8 to 12)	18 (17 to 20)	247 (223 to 270)	−43·1% (−48·1 to −38·1)	407 (368 to 446)	−46·7% (−51·3 to −42·0)	221 (198 to 243)	−58·6% (−62·5 to −54·8)
Hypertensive heart disease	30 (30 to 32)	19 (22 to 29)	218 (122 to 250)	45·1% (−7·6 to 62·4)	360 (201 to 412)	36·0% (−13·4 to 52·2)	127 (79 to 143)	−21·1% (−44·5 to −12·5)
Blindness and vision impairment	28 (25 to 29)	20 (19 to 23)	215 (149 to 294)	28·0% (24·3 to 32·2)	354 (245 to 486)	20·0% (16·5 to 23·9)	199·7 (135 to 282)	−10·6% (−13·3 to −7·9)

Data in parentheses are 95% uncertainty intervals. DALYs=disability-adjusted life-years.

The fifth leading cause of DALYs was headache disorders, in this case exclusively to non-fatal diseases (in GBD, headache disorders are only counted in terms of disability, not mortality). DALYs caused by road injuries decreased by 53·4% (95% UI −57·2 to −49·5) between 1990 and 2017. Significant reductions were also seen in DALYs caused by stroke; tracheal, bronchus, and lung cancer; stomach cancer; cirrhosis and other chronic liver diseases; neonatal disorders and congenital birth defects; self-harm; and cardiomyopathy and myocarditis (figure 2). All these causes also decreased significantly during this period in terms of age-standardised DALY rates (table 3; full data for DALY rates are available online, suggesting that these reductions reflect an actual health improvement for these causes not only for the population as a whole, but also for specific age groups.

The largest increases in number of DALYs (table 3) from 1990 to 2017 were seen for Alzheimer's disease and other dementias (77·9%), age-related hearing loss (54·8%), and hypertensive heart disease (45·1%). How-ever, age-standardised DALY rates decreased slightly for both Alzheimer's disease and other dementias (percentage change −13·8%, 95% UI −18·4 to −9·1) and hypertensive heart diseases (−21·1%, −44·5 to −12·5), while age-related hearing loss had no change (0·2%, −2·0 to 2·3). Even if not in the top 20 causes by number of DALYs, an increase of 39·7% (28·4 to 51·7) was seen in DALYs due to pancreatic cancer (from 151 000 DALYs in 1990 to 196 000 DALYs in 2017), and of 164·7% (129·7 to 202·5) was seen for DALYs due to uterine cancer (from 13 100 to 34 600 DALYs). For neoplasms (Level 2), the age-standardised DALY rate decreased by 32·5% (−28·7 to −36·4%) from 1990 to 2017, with the number of DALYs staying almost the same (3·32 million in 1990 and 3·20 million in 2017).

For deaths and DALYs attributable to risk factors, the percentage of DALYs and the number of deaths for the top 20 risk factors in 2017 by Level 2 causes are shown in the appendix (pp 3–4).

Smoking was the main cause of DALYs and was associated particularly with neoplasms and cardiovascular diseases. High-fasting plasma glucose concentration)^18^ was the second leading risk factor for DALYs, associated with diabetes and kidney diseases and cardiovascular diseases.

The third leading risk factor was high systolic blood pressure, the main cause of cardiovascular diseases. In terms of number of deaths, high blood pressure was the top attributable risk factor, followed by high fasting plasma glucose and smoking. High body-mass index (BMI) was the fourth leading attributable risk factor for both number of DALYs and number of deaths.

Potentially modifiable behaviours and metabolic risks—also associated with modifiable behaviours—have a major role in risk of mortality and DALYs. Among the top 15 risk factors for DALYs, eight are behavioural risks (eg, smoking, alcohol use, and diet low in whole grains) and three are metabolic risks that are closely linked with behaviours (eg, high BMI; appendix pp 3–4). These risk factors affect deaths and DALYs due to cardiovascular diseases, neoplasms, diabetes and kidney diseases, lower respiratory infections, and liver diseases.

Of 181 000 deaths (95% UI 171 000 to 190 000) due to cancer in 2017, 44 400 (41 200 to 47 800) were attributable to smoking, 12 000 (9600 to 14 800) to alcohol use, and 9500 (5400 to 14 200) to high BMI. Of 217 000 deaths (207 000 to 227 000) in 2017 casued by cardiovascular disease, 109 900 (92 700 to 126 800) were attributed to high systolic blood pressure, 47 600 (31 100 to 65 700) to high LDL cholesterol, 28 700 (19 700 to 38 500) to diet low in whole grains, and 15 900 (8500 to 24 900) to low physical activity.

When combined, dietary risks were the third leading risk factor for deaths in 2017, after high blood pressure and high fasting plasma glucose concentration; and the fourth for DALYs after tobacco consumption (eg, chewing), high fasting plasma glucose concentration, and high blood pressure. Dietary risks are a cause of cardiovascular diseases, neoplasms, diabetes, and kidney diseases. Among the top ten attributable Level 3 risk factors for DALYs in 2017, five decreased signficantly between 1990 and 2017 (alcohol use, −58·0%, [95% UI −73·9 to −38·1]; high LDL cholesterol, −40·1% [–47·0 to −33·6]; high systolic blood pressure, −34·1% [–38·2 to −30·1]; smoking, −30·3% [–35·0 to −25·6]; and diet low in whole grains, −28·5% [–34·7 to −21·7]; full data on risk factors for DALYs are available online), while six did not vary signficantly (high fasting plasma glucose concentration, high BMI, diet low in whole grains, particulate matter pollution, impaired kidney function, and drug use; data not shown). For some risk factors, variations over time were not significant due to wide UIs, mainly attributable to the lack of sufficient evidence. For example, diet high in sugar-sweetened beverages is responsible for an average, but non-significant decrease of 12·2% (95% UI −43·5 to 83·6) in attributable number of DALYs.

When comparing the burden of disease among the EU15 countries, Italy's ranking improved between 1990 and 2017 in terms of both YLLs and YLDs (appendix pp 5–6), and globally (appendix pp 11–12). In 2017, Italy had the fifth lowest age-standardised death rate in the world (first lowest in the EU15), an improvement of ten positions since 1990. Italy had the fourth lowest age-standardised YLL rate in the world in 2017 (first lowest in the EU15; an improvement from tenth lowest globally in 1990) and the 48th lowest age-standardised YLD rate (third in the EU15; an improvement from 50th lowest globally in 1990).

In the ranking of the main causes of age-standardised YLLs (appendix p 5), in Italy, lower respiratory infections, chronic obstructive pulmonary disease (COPD), prostate cancer, and self-harm ranked better than the avergae of the other EU15 countries, while hypertensive heart disease, liver cancer, stomach cancer, and diabetes (both type 1 and 2; data not shown) ranked worse than the avergae of the other EU15 countries.

Compared with the other EU15 countries, in 2017 Italy had significantly lower age-standardised DALY rates due to self-harm, COPD, and cirrhosis and other chronic liver diseases and did not have significantly worse rates than any of the other EU15 countries for any diseases or injuries in Italy's top 25 causes of DALYs (appendix p 6). Out of the EU15 countries, Italy had the highest age-standardised DALY rate for sense organ diseases (including blindness and vision impairment, and age-related and other hearing loss) and for other musculoskeletal disorders (inferred from data in the appendix [p 6]). With respect to life expectancy at birth Italy ranked first, and for HALE at birth it ranked second among all the EU15 countries in 2017 (table 4), and it ranked third for life expectancy at birth and fourth for HALE at birth globally (data not shown).

**Table 4:** Age-standardised life expectancy and HALE at birth and at age 65 years in 1990 and 2017, both sexes combined, for Italy and selected western European countries (EU15)

	**Life expectancy**				**HALE**			
	At birth		At age 65 years		At birth		At age 65 years	
	1990	2017	1990	2017	1990	2017	1990	2017
Austria	75·8 (75·7–75·9)	81·8 (81·3–82·2)	16·6 (16·5–16·6)	20·2 (19·8–20·5)	66·0 (63·2–68·5)	70·4 (67·2–73·3)	12·6 (11·4–13·6)	15·1 (13·7–16·4)
Belgium	76·0 (76·0–76·1)	81·4 (80·9–81·8)	16·6 (16·5–16·6)	20·1 (19·7–20·4)	65·9 (63·0–68·4)	69·6 (66·2–72·5)	12·4 (11·3–13·4)	14·8 (13·3–16·1)
Denmark	75·0 (74·9–75·1)	80·8 (80·2–81·3)	16·1 (16·0–16·1)	19·4 (19·0–19·7)	65·3 (62·4–67·7)	69·6 (66·4–72·4)	12·1 (11·0–13·1)	14·5 (13·2–15·7)
Finland	75·1 (75·0–75·2)	81·4 (80·9–81·9)	16·2 (16·2–16·3)	20·1 (19·7–20·4)	64·9 (62·0–67·5)	69·8 (66·4–72·7)	12·2 (11·1–13·2)	15·0 (13·6–16·3)
France	77·0 (77·0–77·1)	82·8 (82·4–83·3)	17·9 (17·9–17·9)	21·6 (21·3–21·9)	67·2 (64·4–69·7)	71·7 (68·6–74·5)	13·6 (12·5–14·7)	16·4 (15·0–17·7)
Germany	75·5 (75·5–75·6)	80·6 (79·7–81·5)	16·3 (16·3–16·3)	19·4 (18·8–20·0)	65·6 (62·7–68·1)	69·5 (66·3–72·5)	12·3 (11·2–13·2)	14·5 (13·1–15·8)
Greece	77·5 (77·4–77·6)	81·0 (80·5–81·5)	17·3 (17·3–17·4)	19·8 (19·5–20·2)	67·3 (64·4–69·9)	69·9 (66·9–72·6)	13·1 (12·0–14·1)	15·0 (13·7–16·2)
Ireland	74·9 (74·7–75·0)	81·8 (81·4–82·3)	15·2 (15·1–15·2)	20·0 (19·6–20·3)	65·2 (62·4–67·6)	70·4 (67·1–73·1)	11·5 (10·5–12·4)	14·9 (13·5–16·2)
Italy	77·1 (77·0–77·1)	83·2 (82·7–83·6)	17·3 (17·2–17·3)	21·0 (20·7–21·3)	67·0 (64·1–69·5)	71·9 (68·7–74·7)	13·1 (11·9–14·1)	15·9 (14·4–17·2)
Luxembourg	75·4 (75·2–75·6)	81·7 (81·0–82·4)	16·3 (16·2–16·4)	19·9 (19·4–20·4)	64·9 (61·9–67·5)	69·7 (66·3–72·6)	12·0 (10·83–0)	14·6 (13·2–15·9)
Netherlands	77·0 (76·9–77·1)	81·5 (81·0–82·0)	16·9 (16·8–16·9)	19·7 (19·4–20·0)	66·7 (63·8–69·3)	70·2 (66·9–73·0)	12·7 (11·6–13·7)	14·7 (13·4–16·0)
Portugal	74·1 (74·1–74·2)	81·4 (81·0–81·9)	15·8 (15·7–15·8)	20·1 (19·8–20·4)	64·2 (61·2–66·6)	70·1 (66·9–73·0)	11·8 (10·7–12·7)	15·0 (13·6–16·3)
Spain	77·0 (77·0–77·1)	83·1 (82·7–83·5)	17·5 (17·5–17·5)	21·2 (20·9–21·5)	67·2 (64·4–69·6)	72·1 (68·9–74·8)	13·3 (12·2–14·3)	16·2 (14·8–17·4)
Sweden	77·7 (77·6–77·8)	82·5 (82·1–82·9)	17·4 (17·3–17·4)	20·4 (20·1–20·7)	67·4 (64·4–69·9)	70·9 (67·6–73·9)	13·2 (12·1–14·2)	15·3 (13·9–16·6)
UK	75·8 (75·7–75·8)	81·0 (80·9–81·1)	16·1 (16·1–16·1)	19·7 (19·6–19·7)	65·7 (62·9–68·2)	69·3 (66·0–72·1)	12·3 (11·2–13·2)	14·7 (13·3–15·9)

Data are years, with 95% uncertainty intervals in parentheses. HALE=healthy life expectancy.

In terms of life expectancy and HALE at age 65 years, in 2017 Italy ranked third highest among the EU15, just after France and Spain, and seventh for life expectancy and eighth for HALE globally (data not shown). In 2017, life expectancy in Italy at age 65 years was 21·0 years (95% UI 20·7–21·3), an increase of more than 3·5 years compared with 1990 (table 4).

Looking at risk factor categories attributed to DALYs, a similar distribution is seen for Italy as for the other EU15 countries (appendix p7). Magnitude and time trends of the burden attributable to the three main Level 1 categories of risk (environmental and occupational, behavioural, metabolic), in terms of DALY rates, are similar between Italy and the average of the EU15 countries (appendix p 8), with slightly higher than average levels of metabolic and environmental risks compensated by lower levels of behavioural risks. Behavioural risks attributable to DALY rates in Italy had consistently less of an effect between 1990 and 2017, while metabolic, environmental, and occupational risks had more of an effect than the average of the GBD region of western Europe (appendix p 8). In particular, the burden on DALY rates attributable to alcohol use decreased in Italy by 60·7% (95% UI −75·6 to −42·0) from 1990 to 2017, whereas in western Europe it decreased by 37·5% (−46·2 to −28·5).

## Discussion

Our analysis of Italy's burden of disease is the first to use the double lens of temporal trends and a comparison with other EU15 countries. The analysis of the GBD 2017 estimates confirm that Italy's main health indicators are among the best in Europe and globally. Life expectancy is high and has shown a substantial and consistent increase since 1990. The overall positive situation is exemplified by a good general lifestyle, with lower than average exposure to behavioural risk factors, and a high HAQ Index—even higher than expected in terms of SDI value.

Italy's reduction in age-standardised mortality for most diseases and very low infant and under-5 mortality should be seen as further, and more specific, indicators of the efficiency of the health-care system. By contrast, the combination of low fertility and a high life expectancy have led to a rapid change in the population structure, with the mean age of the population increasing from 39 years in 1990 to 45 years in 2017. The consequences of this trend, in terms of an epidemiological shift, are already visible on the disease burden. Our analysis of rates of deaths, YLLs, YLDs, and DALYs shows an increase between 1990 and 2017 for several diseases, risk factors, and injuries, accompanied by a general reduction in age-standardised rates.

This phenomenon is mainly the consequence of improvement in individual health conditions as well as an increased overall burden due to population ageing. For example, neoplasms, despite a 32·5% decrease in age-standardised DALY rates over the study period, have remained stable in terms of the number of DALYs, with a 68·2% increase in YLDs balanced by a 5·9% decrease in YLLs, attributable to better survival rates in 1990 than in 2017.

However, the absolute increasing rates of some diseases are a cause for concern and require further in-depth investigation of both causes and consequences to better direct health service resources. For instance, Alzheimer's disease and other dementias, which had increased YLLs, YLDs, deaths, and DALYs, increased the GBD rankings, placing Italy at the highest rates of both YLLs and YLDs among the EU15 countries.

Other examples are pancreatic cancer, for which the number of deaths has increased by 65·6%, and uterine cancer for which the number of deaths has increased by 177% between 1990 and 2017.

Italy's DALY burden attributable to lifestyle-dependent risk factors of alcohol use, smoking, high systolic blood pressure, and high LDL cholesterol has decreased significantly. DALYs attributable to high BMI and high fasting plasma glucose concentration did not vary significantly since 1990, suggesting that primary prevention and health promotion have been insufficient to tackle these issues. For the factors listed above, the trends are in line with the average for western Europe, with Italy having a slightly lower attributable burden—with the exception of alcohol use, for which the reduction in Italy has been more substantial than for other EU15 countries.

The reduction in deaths due to road injuries indicates that preventive measures related to driving regulations and enforcement, road network efficiency, and effective response from emergency services have had a positive effect on road safety. Drug use disorders have also substantially decreased in terms of number of deaths (−35·4%), YLLs (−62·5%), and YLDs (−11·0%), likely due in part to the introduction of effective awareness-raising drug-related policies.

However, drug use disorders were still responsible for 147 000 DALYs in 2017. Increased efforts in health promotion and prevention interventions are required to address the main risk factors for lifestyle-dependent diseases. Smoking, high fasting plasma glucose concentration, high blood pressure, dietary risks, high BMI, and alcohol use are still the main risk factors of Italy's disease burden. Margins for increased investments in prevention can be seen if we consider that in Italy in 2015 the health system spent €87 per capita on preventive measures, against €111 in Germany and €155 in the UK.^22^

Despite Italy being an interesting example of the results that can be achieved by a mix of relatively healthy lifestyles and a universal health system, two main issues require attention. First, the combination of low fertility and high life expectancy are contributing to population ageing and its consequences on the change in disease burden. Second, public health spending has reduced and out-of-pocket expenditure has increased, suggesting a shift of essential costs from the public to individual families.^23^, ^24^

If we consider the relevant disparities existing in Italy, in terms of health outcomes, system performance, and GDP, with better outcomes and performance in the north than in the south,^25^ the main limitation of this analysis is the unavailability of estimates at subnational level. Our future analyses will look at subnational level estimates. In this Article we did not do a comprehensive comparison between GBD estimates for Italy and Italian data; however, this comparative analysis is being done and is among the objectives of the Italian GBD Initiative.

GBD estimates are an important resource to drive evidence-based planning and their use can further strengthen the thorough analyses already undertaken by several groups of experts.^23^, ^25^, ^26^, ^27^ Subnational GBD estimates will further strengthen their value and help analyse regional differences in the distribution of the burden and in the provision of care.
