# Supplementary material for: Italy's health performance, 1990–2017: findings from the Global Burden of Disease Study 2017
Source: Lancet Public Health. 2019 Nov 20;4(12):e645–57. doi: 10.1016/S2468-2667(19)30189-6 (PMC7098474; doi:10.1016/S2468-2667(19)30189-6)
Supplement: Supplementary appendix [file mmc1.pdf]

THE LANCET
Public Health

Supplementary appendix

This appendix formed part of the original submission and has been peer reviewed. We post it as supplied by the authors.

Supplement to: GBD 2017 Italy Collaborators. Italy's health performance, 1990–2017: findings from the Global Burden of Disease Study 2017. *Lancet Pubic Health* 2019; published online Nov 20. https://doi.org/10.1016/S2468-2667(19)30206-3.

Appendix to “Italy’s health performance, 1990–2017: Findings from the Global Burden of Disease Study 2017”

Supplementary Figure 1. Health Access and Quality Index of top twelve performing countries in 1990 and 2017: time trend 1990-2017

Supplementary Figure 2a. Percentage of disability-adjusted life-years (DALYs) by 22 Level 3 causes related to 20 main Level 3 risk factors in Italy in 2017, for both sexes combined

Supplementary Figure 2b. Number of deaths (in thousands) by 22 Level 2 causes related to 20 main Level 3 risk factors in Italy in 2017, for both sexes combined

Supplementary Figure 3. Ranking of age-standardised years of life lost (YLLs), Level 3, in 2017, for Italy and selected western European countries (EU15)

Supplementary Figure 4. Ranking of age-standardised disability-adjusted life-years (DALYs), Level 3, in 2017, for Italy and selected western European countries (EU15)

Supplementary Figure 5. Level 2 risk factor categories attributed to disability-adjusted life-years (DALYs), in 2017, in percentage of total DALYs, for selected western European countries (EU15)

Supplementary Figure 6. Level 1 risk factor categories attributed to disability-adjusted life-years (DALYs), time trend 1990–2017, for Italy and Western Europe

Supplementary Table 1. Number of deaths by Level 3 causes, death rates, and age-standardised death rates for the year 2017, and median percentage change between 1990 and 2017, ordered by ranking in number of deaths in 2017 (top 20 causes)

Supplementary Table 2. Prevalence by Level 3 causes, prevalence rates, and age-standardised prevalence rates for the year 2017, and median percentage change between 1990 and 2017, ordered by ranking in prevalence in 2017 (top 20 causes)

Supplementary Table 3a. Age-standardised rates of death and years of life lost due to premature mortality (YLLs) in 1990 and 2017, both sexes combined, with 95% uncertainty intervals, for Italy and selected western European countries (EU15); countries in alphabetical order

Supplementary Table 3b. Age-standardised rates of years lived with disability (YLDs) and disability-adjusted life-years (DALYs) in 1990 and 2017, both sexes combined, with 95% uncertainty intervals for Italy and selected western European countries (EU15); countries in alphabetical order

Supplementary Figure 1. Health Access and Quality Index of the top twelve performing countries in both 1990 and 2017: time trend 1990-2017

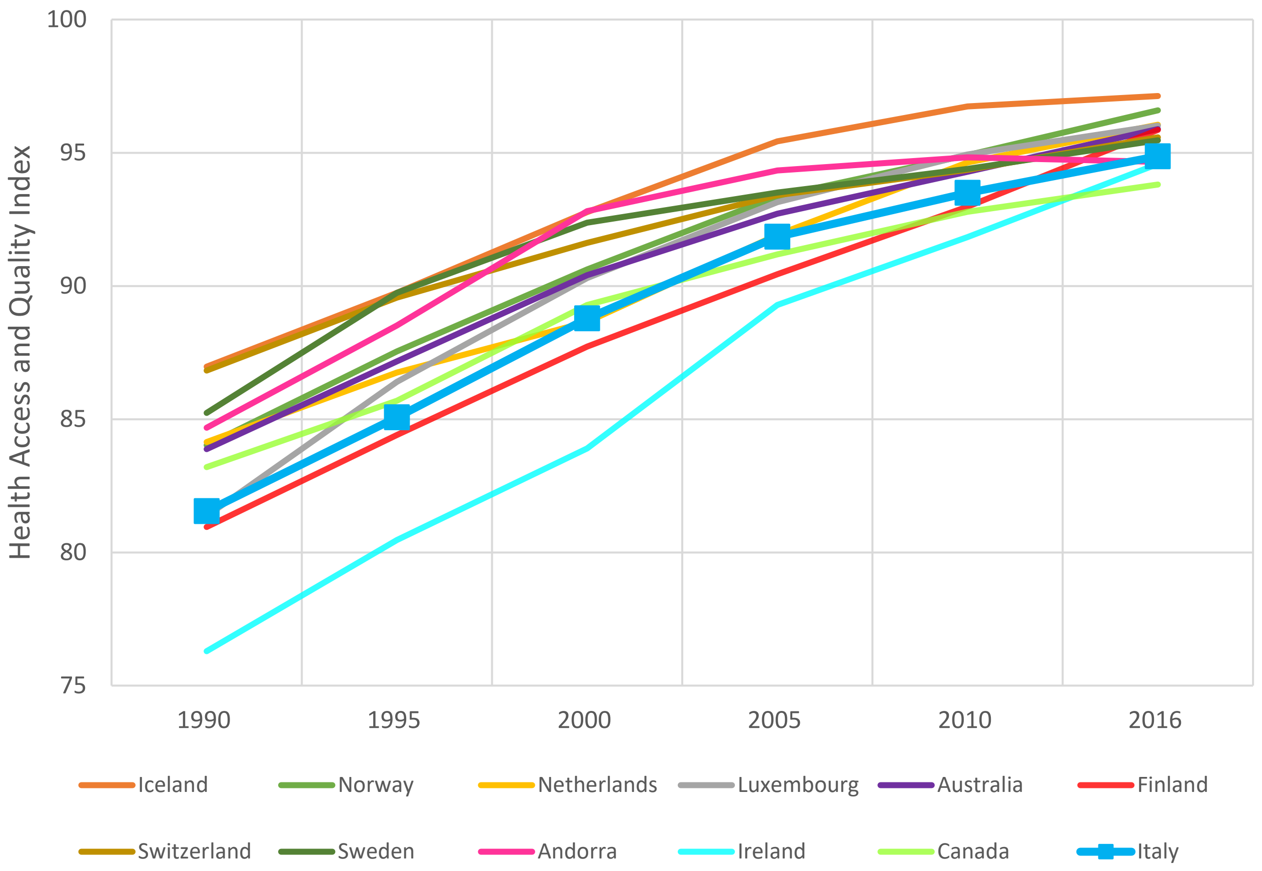

Supplementary Figure 2a. Percentage of disability-adjusted life-years (DALYs) by 22 Level 3 causes related to 20 main Level 3 risk factors in Italy in 2017, for both sexes combined

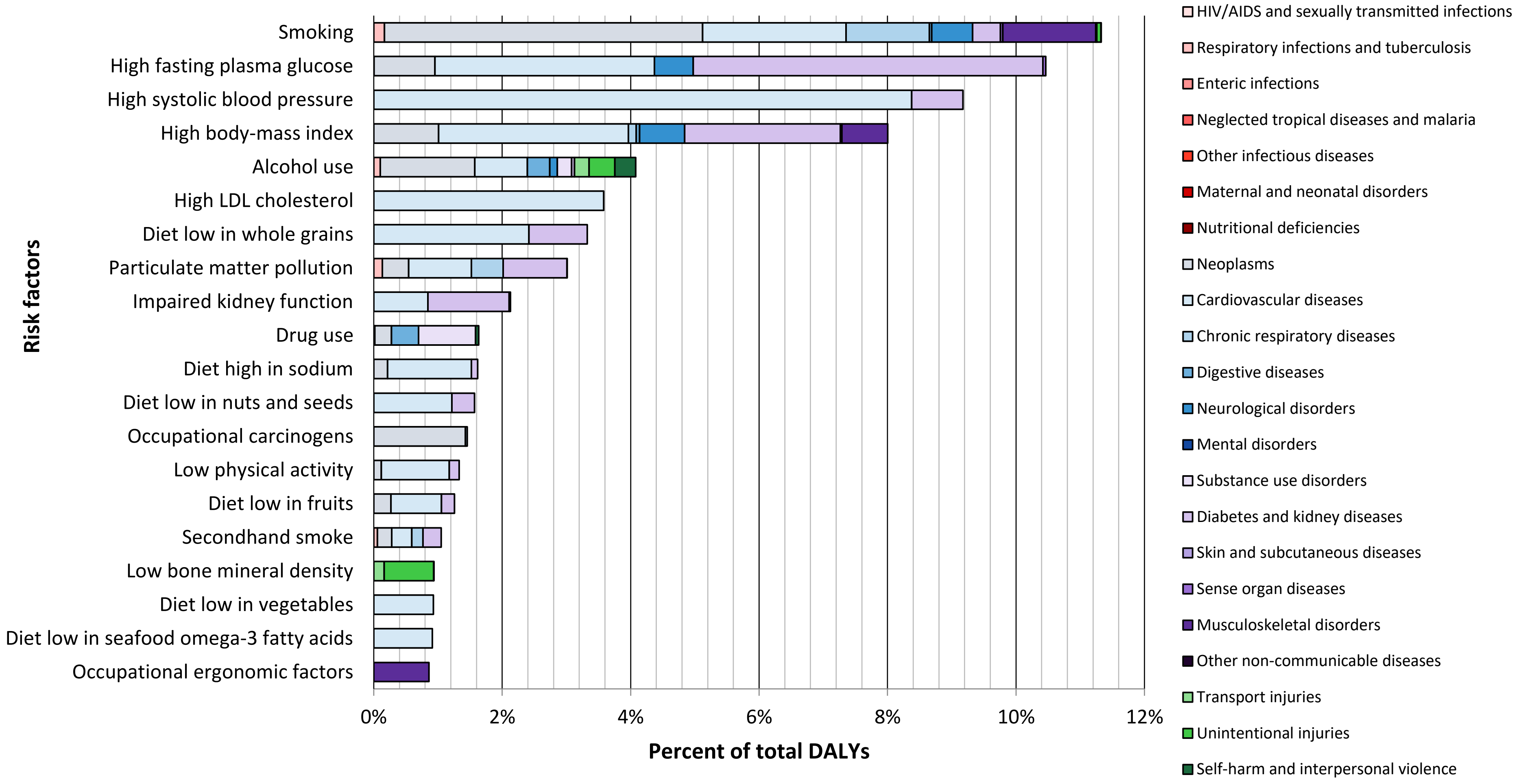

Supplementary Figure 2b. Number of deaths (in thousands) by 22 Level 2 causes related to 20 main Level 3 risk factors in Italy in 2017, for both sexes combined

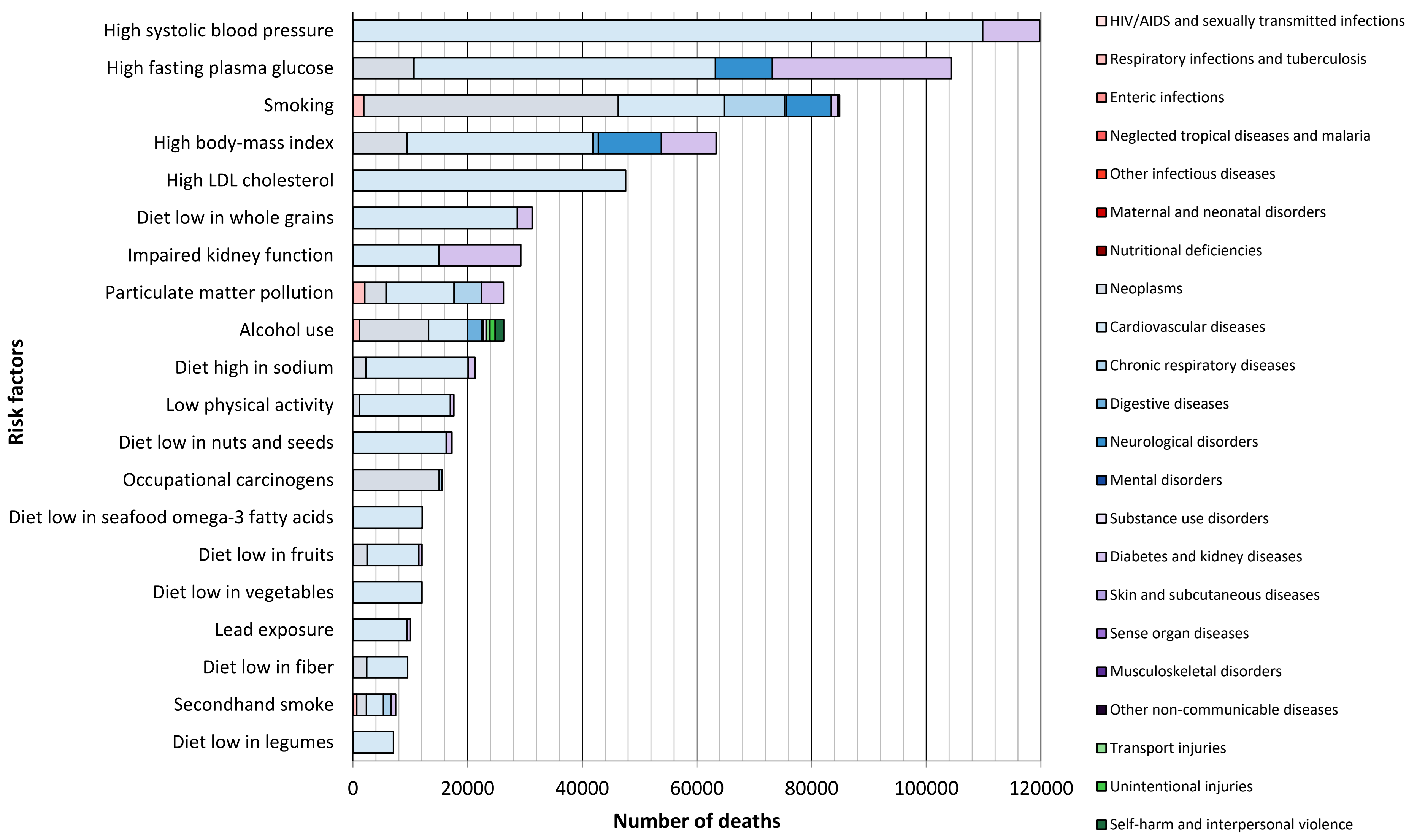

Supplementary Figure 3. Ranking of age-standardised years of life lost (YLLs), Level 3, in 2017, for Italy and selected western European countries (EU15)

	Austria	Belgium	Denmark	Finland	France	Germany	Greece	Ireland	**Italy**	Luxembourg	Netherlands	Portugal	Spain	Sweden	United Kingdom
Ischemic heart disease	1	1	1	1	2	1	1	1	1	1	2	2	1	1	1
Tracheal, bronchus, and lung cancer	2	2	2	5	1	2	2	2	2	2	1	3	2	3	2
Stroke	5	4	4	4	5	4	3	4	3	3	4	1	3	4	3
Alzheimer's disease and other dementias	6	7	8	3	6	7	6	5	4	5	8	6	4	5	6
Neonatal disorders	7	8	7	16	4	5	5	9	5	14	5	10	6	7	4
Road injuries	11	6	15	10	7	12	4	13	6	8	13	7	10	13	14
Colon and rectum cancer	9	10	6	9	8	8	11	7	7	7	6	5	5	6	9
Breast cancer	12	12	11	12	10	10	8	10	8	10	9	13	12	8	10
Self-harm	3	3	5	2	3	3	18	3	9	4	3	9	8	2	7
Cirrhosis and other chronic liver diseases	4	11	10	6	9	6	17	15	10	6	22	8	9	16	11
Congenital birth defects	10	13	12	13	11	11	7	8	11	26	10	15	11	10	12
Pancreatic cancer	13	14	16	11	12	13	14	14	12	11	12	19	14	12	15
Diabetes mellitus	15	24	14	27	21	16	26	26	13	25	20	14	21	18	32
Chronic obstructive pulmonary disease	8	5	3	15	20	9	10	6	14	9	7	12	7	9	5
Stomach cancer	22	22	23	24	23	19	16	19	15	23	17	11	15	31	23
Liver cancer	23	26	27	25	14	28	19	30	16	22	32	21	18	29	28
Leukaemia	18	16	18	19	15	20	15	17	17	15	15	20	17	19	18
Brain and nervous system cancer	19	17	19	18	18	18	12	16	18	13	16	17	16	17	17
Lower respiratory infections	29	9	13	30	13	14	9	11	19	12	11	4	13	14	8
Other malignant neoplasms	20	18	22	21	19	21	22	21	20	19	19	23	19	20	21
Hypertensive heart disease	21	59	61	23	54	26	28	53	21	42	55	41	39	39	42
Chronic kidney disease	16	23	21	37	34	17	13	23	22	21	24	18	20	28	35
Non-Hodgkin lymphoma	28	29	30	22	26	31	33	22	23	28	23	24	27	23	24
Endocrine, metabolic, blood, and immune disorders	14	28	24	41	25	29	38	27	24	29	29	26	26	41	29
Prostate cancer	27	19	20	20	24	23	21	18	25	24	18	22	24	15	19

Supplementary Figure 4. Ranking of age-standardised disability-adjusted life-years (DALYs), Level 3, in 2017, for Italy and selected western European countries (EU15)

	Austria	Belgium	Denmark	Finland	France	Germany	Greece	Ireland	**Italy**	Luxembourg	Netherlands	Portugal	Spain	Sweden	United Kingdom
Low back pain	1	1	1	2	1	1	2	1	1	1	1	1	1	1	1
Headache disorders	3	2	4	3	2	3	3	3	2	2	2	2	2	3	3
Ischemic heart disease	2	3	3	1	5	2	1	2	3	3	4	5	3	2	2
Diabetes mellitus	7	9	2	7	14	12	11	5	4	4	6	3	7	6	12
Depressive disorders	9	5	11	5	4	6	7	4	5	8	8	6	5	4	6
Neonatal disorders	4	11	8	4	8	4	8	9	6	7	5	7	6	9	4
Anxiety disorders	8	12	12	19	6	7	10	8	7	12	9	9	9	11	15
Tracheal, bronchus, and lung cancer	6	4	5	15	3	5	5	7	8	5	3	10	4	14	7
Neck pain	13	14	9	10	13	11	13	12	9	11	11	8	11	12	11
Stroke	12	10	7	9	11	9	4	10	10	9	10	4	8	7	8
Falls	5	6	10	6	7	10	14	11	11	6	12	14	10	5	10
Road injuries	16	13	19	18	10	17	6	20	12	14	20	12	14	20	26
Alzheimer's disease and other dementias	15	15	15	11	12	14	15	15	13	13	13	13	13	16	14
Congenital birth defects	14	16	14	17	15	15	12	13	14	26	14	18	15	13	13
Age-related and other hearing loss	19	17	25	21	16	20	16	21	15	22	19	20	17	21	20
Other musculoskeletal disorders	21	23	21	22	17	22	18	19	16	25	17	21	18	23	21
Chronic obstructive pulmonary disease	10	8	6	16	26	8	9	6	17	10	7	11	12	10	5
Colon and rectum cancer	23	21	16	26	20	19	21	17	18	19	16	15	16	19	23
Drug use disorders	18	26	18	13	22	24	23	16	19	18	27	33	19	17	9
Breast cancer	25	19	24	25	19	21	17	22	20	21	18	25	24	24	22
Cirrhosis and other chronic liver diseases	17	20	22	14	18	16	28	28	21	17	35	19	20	29	25
Self-harm	11	7	17	8	9	13	31	14	22	15	15	22	21	8	17
Dermatitis	34	27	23	20	21	29	37	25	23	23	23	28	29	15	19
Blindness and vision impairment	32	29	49	36	49	32	43	29	24	32	49	29	26	77	30
Oral disorders	22	18	20	24	27	23	19	23	25	24	21	26	22	22	27

Supplementary Figure 5. Level 2 risk factor categories attributed to disability-adjusted life-years (DALYs), in 2017, in percentage of total DALYs, for selected western European countries (EU15)

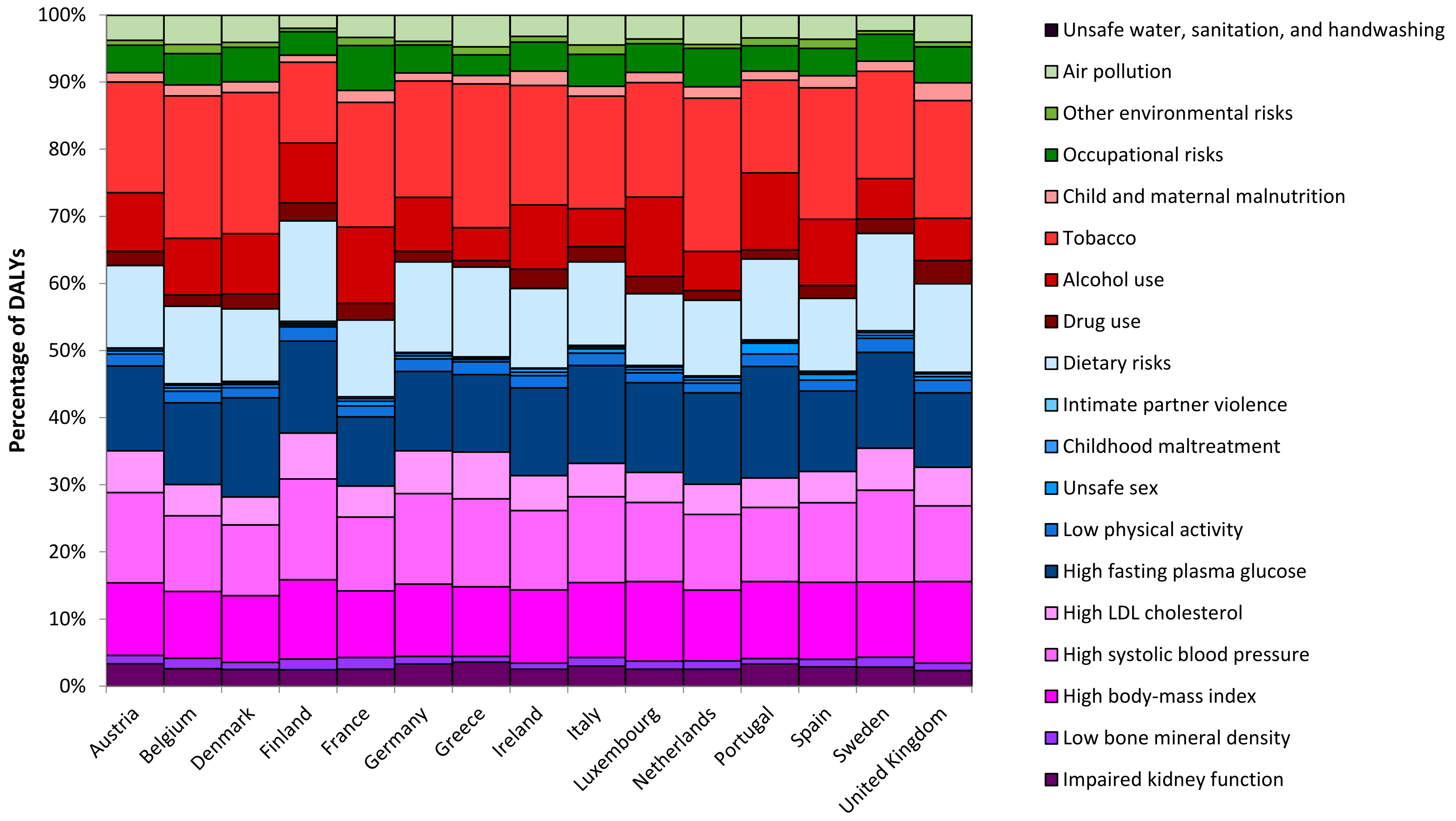

Supplementary Figure 6. Level 1 risk factor categories attributed to disability-adjusted life-years (DALYs), time trend 1990–2017, for Italy and Western Europe*

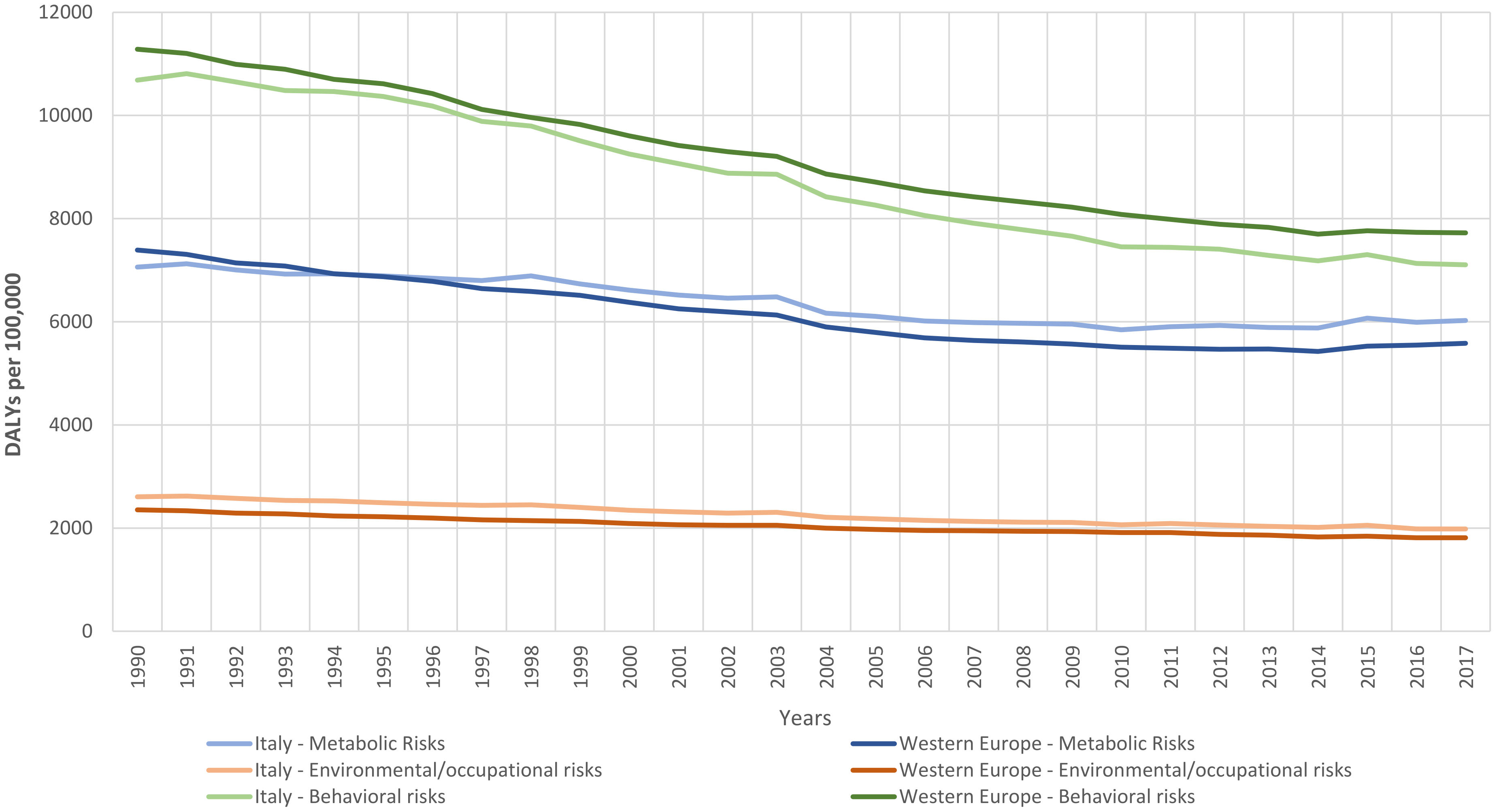

* Western Europe comprises Andorra, Austria, Belgium, Cyprus, Denmark, Finland, France, Germany, Greece, Iceland, Ireland, Israel, Italy, Luxembourg, Malta, Netherlands, Norway, Portugal, Spain, Sweden, Switzerland, United Kingdom.

Supplementary Table 1. Number of deaths by Level 3 causes, death rates, and age-standardised death rates in 2017, and median percentage change between 1990 and 2017, ordered by ranking in number of deaths in 2017 (top 20 causes)

	Rank by number of deaths in 2017		***Deaths (in thousands) (95% UI)***	***Death rate per 100 000 people (95% UI)***	***Age-standardised death rate per 100 000 people (95% UI)***	***Median change between 1990 and 2017, %***		
	1990	2017	2017	2017	2017	***Deaths (95% UI)***	***Death rate (95% UI)***	***Age-standardised death rate (95% UI)***
All causes			622·1 (594·4 – 649·3)	1026·7 (980·9 – 1071·6)	368·0 (351·7 – 385·6)	14·9 (9·9 – 19·8)	7·7 (3·1 – 12·3)	-41·3 (-43·9 – -38·7)
Ischaemic heart disease	1 (1 – 1)	1 (1 – 1)	96·5 (89·8 – 107·3)	159·2 (148·2 – 177·0)	51·4 (47·9 – 56·6)	-6·8 (-12·7 – 0·7)	-12·6 (-18·2 – -5·6)	-55·2 (-58·0 – -51·8)
Alzheimer's disease and other dementias	3 (3 – 3)	2 (2 – 2)	73·3 (69·0 – 77·5)	121·0 (113·8 – 127·9)	34·5 (32·4 – 36·5)	117·7 (104·7 – 129·5)	104·0 (91·9 – 115·2)	-12·6 (-17·9 – -7·6)
Stroke	2 (2 – 2)	3 (3 – 3)	59·4 (55·2 – 66·3)	97·9 (91·0 – 109·3)	30·4 (28·3 – 33·7)	-11·7 (-17·6 – -4·3)	-17·3 (-22·8 – -10·3)	-58·9 (-61·8 – -55·7)
Tracheal, bronchus, and lung cancer	4 (4 – 4)	4 (4 – 4)	34·1 (31·9 – 36·3)	56·3 (52·7 – 60·0)	23·5 (22·0 – 25·1)	6·3 (-0·8 – 13·8)	-0·3 (-7·0 – 6·7)	-32·8 (-37·4 – -28·0)
Chronic obstructive pulmonary disease	6 (5 – 6)	5 (5 – 5)	25·7 (23·9 – 27·6)	42·3 (39·5 – 45·6)	13·1 (12·3 – 14·2)	35·0 (24·6 – 48·0)	26·6 (16·8 – 38·8)	-35·5 (-40·6 – -29·1)
Hypertensive heart disease	13 (13 – 18)	6 (6 – 17)	22·0 (7·4 – 26·0)	36·3 (12·2 – 43·0)	10·6 (4·1 – 12·4)	122·6 (12·3 – 154·5)	108·6 (5·3 – 138·5)	-2·9 (-45·4 – 9·8)
Colon and rectum cancer	10 (9 – 10)	7 (6 – 7)	21·0 (19·5 – 22·6)	34·6 (32·2 – 37·3)	13·3 (12·3 – 14·3)	30·1 (20·3 – 40·1)	21·9 (12·8 – 31·3)	-24·2 (-29·8 – -18·2)
Diabetes mellitus	9 (8 – 9)	8 (7 – 8)	18·6 (17·3 – 19·9)	30·6 (28·5 – 32·8)	10·5 (9·8 – 11·2)	12·2 (3·2 – 21·3)	5·2 (-3·2 – 13·7)	-40·5 (-45·1 – -35·8)
Chronic kidney disease	15 (14 – 15)	9 (8 – 9)	14·3 (13·3 – 15·3)	23·6 (22·0 – 25·3)	7·3 (6·8 – 7·9)	74·2 (61·2 – 89·2)	63·2 (51·1 – 77·3)	-19·5 (-25·3 – -12·9)
Lower respiratory infections	17 (16 – 18)	10 (9 – 10)	13·2 (12·1 – 14·2)	21·7 (20·0 – 23·5)	7·1 (6·6 – 7·7)	86·0 (70·9 – 102·0)	74·4 (60·2 – 89·4)	-18·9 (-25·6 – -11·9)
Breast cancer	11 (11 – 11)	11 (10 – 12)	13·0 (11·8 – 14·2)	21·5 (19·5 – 23·4)	9·2 (8·3 – 10·0)	11·2 (0·7 – 21·1)	4·2 (-5·6 – 13·5)	-32·5 (-39·0 – -26·3)
Pancreatic cancer	16 (15 – 16)	12 (11 – 13)	12·7 (11·8 – 13·7)	20·9 (19·4 – 22·6)	8·4 (7·8 – 9·1)	65·6 (53·1 – 79·1)	55·2 (43·5 – 67·9)	1·2 (-6·7 – 9·6)
Stomach cancer	7 (6 – 7)	13 (12 – 14)	12·1 (11·3 – 13·0)	20·0 (18·6 – 21·4)	7·7 (7·2 – 8·3)	-32·1 (-37·2 – -27·1)	-36·4 (-41·1 – -31·7)	-60·0 (-62·9 – -57·1)
Cirrhosis and other chronic liver diseases	8 (7 – 8)	14 (13 – 15)	11·7 (10·6 – 12·7)	19·3 (17·5 – 21·0)	8·2 (7·4 – 9·0)	-33·9 (-39·7 – -27·9)	-38·0 (-43·5 – -32·4)	-59·1 (-62·8 – -55·3)
Liver cancer	14 (13 – 14)	15 (14 – 17)	10·6 (9·6 – 11·7)	17·5 (15·9 – 19·3)	7·1 (6·4 – 7·9)	17·3 (6·9 – 28·9)	9·9 (0·2 – 20·8)	-26·9 (-33·6 – -19·5)
Atrial fibrillation and flutter	24 (22 – 23)	16 (15 – 16)	9·9 (9·1 – 11·9)	16·4 (15·1 – 19·7)	4·8 (4·4 – 5·8)	106·4 (92·7 – 119·4)	93·4 (80·6 – 105·7)	-13·0 (-19·0 – -7·4)
Prostate cancer	19 (17 – 22)	17 (11 – 16)	9·7 (8·2 – 14·2)	16·0 (13·5 – 23·5)	5·4 (4·6 – 8·2)	55·7 (36·6 – 97·3)	46·0 (28·0 – 84·9)	-16·7 (-27·5 – 8·5)
Cardiomyopathy and myocarditis	5 (5 – 10)	18 (18 – 21)	7·8 (6·8 – 8·7)	12·9 (11·2 – 14·3)	4·1 (3·7 – 4·5)	-64·5 (-68·7 – -53·7)	-66·7 (-70·7 – -56·6)	-84·0 (-85·9 – -77·9)
Falls	18 (17 – 19)	19 (19 – 20)	7·7 (7·1 – 8·3)	12·6 (11·7 – 13·7)	4·2 (3·9 – 4·5)	19·1 (8·6 – 30·9)	11·6 (1·8 – 22·7)	-45·4 (-50·0 – -40·4)
Bladder cancer	20 (19 – 20)	20 (19 – 20)	7·6 (7·0 – 8·4)	12·6 (11·5 – 13·8)	4·4 (4·0 – 4·8)	27·6 (16·6 – 39·8)	19·6 (9·3 – 31·1)	-29·9 (-35·8 – -23·3)

Supplementary Table 2. Prevalence by Level 3 causes, prevalence rates, and age-standardised prevalence rates in 2017, and median percentage changes between 1990 and 2017, ordered by ranking in prevalence in 2017 (top 20 causes)

	Rank by prevalent cases in 2017		***Prevalent cases (in thousands) (95% UI)***	***Prevalence rate per 100 000 people (95% UI)***	***Age-standardised prevalence rate per 100 000 people (95% UI)***	***Median change between 1990 and 2017, %***		
	1990	2017	2017	2017	2017	***Prevalent cases (95% UI)***	***Prevalence rate (95% UI)***	***Age-standardised prev. rate (95% UI)***
All causes			58166·0 (58010·3 – 58334·2)	95987·5 (95730·6 – 96265·0)	92343·9 (91842·3 – 92850·4)	7·0 (6·7 – 7·2)	0·3 (0·0 – 0·5)	-1·0 (-1·5 – -0·6)
Oral disorders	1 (1 – 1)	1 (1 – 2)	27182·7 (25513·9 – 28995·7)	44857·8 (42103·8 – 47849·6)	39795·0 (36807·8 – 42733·9)	-0·8 (-4·2 – 2·9)	-7 (-10·2 – -3·5)	-11·1 (-14·2 – -7·6)
Headache disorders	2 (2 – 2)	2 (1 – 2)	27171·0 (26032·8 – 28464·4)	44838·4 (42960·2 – 46972·9)	41286·6 (39426·9 – 43173·7)	1·7 (-1·1 – 4·5)	-4·7 (-7·3 – -2·1)	-3·4 (-5·8 – -1·0)
Haemoglobinopathies and haemolytic anaemias	3 (3 – 3)	3 (3 – 3)	14919·2 (14517·5 – 15321·7)	24620·2 (23957·2 – 25284·4)	24353·6 (23657·3 – 25011·1)	6·6 (4·0 – 8·8)	-0·1 (-2·5 – 2·0)	-0·5 (-2·9 – 1·6)
Age-related and other hearing loss	5 (5 – 6)	4 (4 – 5)	13977·5 (13620·9 – 14361·8)	23066·1 (22477·6 – 23700·3)	11938·0 (11497·3 – 12352·1)	43·1 (41·5 – 44·8)	34·1 (32·6 – 35·7)	0·9 (-0·1 – 2·0)
Cirrhosis and other chronic liver diseases	4 (4 – 4)	5 (4 – 5)	13771·9 (13230·2 – 14394·4)	22726·8 (21833·0 – 23754·1)	16280·1 (15585·8 – 17011·4)	32·8 (29·4 – 36·0)	24·5 (21·3 – 27·5)	10·8 (7·9 – 13·5)
Upper digestive system diseases	8 (8 – 8)	6 (6 – 7)	9500·0 (8595·8 – 10436·1)	15677·1 (14185·0 – 17221·9)	11217·4 (10088·2 – 12459·2)	17·8 (14·4 – 20·6)	10·4 (7·3 – 13·0)	-1·1 (-2·2 – -0·1)
Low back pain	7 (7 – 7)	7 (6 – 7)	9389·9 (8447·7 – 10463·2)	15495·6 (13940·6 – 17266·7)	11294·4 (10163·6 – 12556·0)	11·7 (5·7 – 17·3)	4·7 (-0·9 – 9·9)	-6·4 (-11·1 – -1·7)
Other skin and subcutaneous diseases	13 (13 – 14)	8 (8 – 10)	8368·5 (8138·3 – 8593·3)	13810·1 (13430·1 – 14181·0)	9144·7 (8914·8 – 9389·0)	42·5 (40·0 – 45·0)	33·6 (31·3 – 35·9)	11·6 (9·9 – 13·4)
Sexually transmitted infections excluding HIV	9 (9 – 9)	9 (9 – 9)	7859·1 (7101·7 – 8707·1)	12969·4 (11719·5 – 14368·7)	10218·8 (9246·0 – 11273·4)	13·2 (8·8 – 17·4)	6·1 (1·9 – 10·0)	-0·3 (-4·1 – 3·2)
Fungal skin diseases	14 (13 – 14)	10 (10 – 11)	7600·2 (6805·5 – 8530·0)	12542·2 (11230·7 – 14076·5)	8132·4 (7313·5 – 9098·8)	32·3 (28·4 – 36·6)	24·1 (20·4 – 28·0)	-1·8 (-2·3 – -1·4)
Tuberculosis	6 (5 – 6)	11 (8 – 12)	7490·1 (6256·2 – 8884·9)	12360·4 (10324·1 – 14662·2)	9938·2 (8404·7 – 11720·6)	-15·9 (-21·4 – -10·6)	-21·2 (-26·3 – -16·2)	-26·3 (-30·5 – -21·7)
Falls	11 (11 – 13)	12 (11 – 12)	7461·5 (6645·7 – 8466·6)	12313·2 (10967·0 – 13971·8)	8110·9 (7053·5 – 9331·1)	19·2 (15·5 – 23·2)	11·7 (8·2 – 15·4)	-9·2 (-11 – -7·4)
Gynaecological diseases	12 (12 – 12)	13 (13 – 15)	6413·8 (5758·2 – 7669·9)	10584·2 (9502·4 – 12657·2)	9490·6 (8721·5 – 10934·3)	6·5 (2·9 – 10·3)	-0·2 (-3·6 – 3·4)	-0·5 (-3·5 – 2·3)
Diabetes mellitus	17 (17 – 18)	14 (14 – 14)	6331·6 (5786·9 – 6973·1)	10448·6 (9549·7 – 11507·3)	5616·9 (5103·0 – 6215·1)	71·6 (54·8 – 94·8)	60·9 (45·1 – 82·6)	26·2 (14·4 – 42·1)
Chronic kidney disease	16 (16 – 16)	15 (15 – 16)	6163·0 (5684·4 – 6714·5)	10170·5 (9380·6 – 11080·6)	5155·8 (4792·5 – 5602·1)	33·6 (27·8 – 40·0)	25·2 (19·8 – 31·3)	-9·2 (-12·8 – -6·1)
Blindness and vision impairment	15 (15 – 15)	16 (13 – 17)	6114·6 (5827·5 – 6464·6)	10090·5 (9616·7 – 10668·0)	6135·4 (5794·8 – 6555·6)	26·7 (23·1 – 30·1)	18·8 (15·4 – 22·0)	-6·4 (-8·7 – -3·8)
Vitamin A deficiency	10 (10 – 10)	17 (16 – 17)	5887·5 (5222·8 – 6582·9)	9715·7 (8618·8 – 10863·3)	12198·2 (10880·8 – 13661·4)	-12·2 (-19·1 – -3·8)	-17·7 (-24·2 – -9·8)	-11·4 (-18·5 – -3·5)
Osteoarthritis	21 (20 – 21)	18 (18 – 18)	4709·1 (4217·3 – 5276·6)	7771·2 (6959·5 – 8707·7)	3646·1 (3265·8 – 4088·3)	52·4 (46·7 – 58·5)	42·8 (37·5 – 48·6)	3·7 (-0·2 – 7·6)
Neck pain	18 (18 – 19)	19 (19 – 19)	4502·9 (3957·5 – 5068·6)	7430·9 (6530·9 – 8364·3)	4824·4 (4225·2 – 5466·1)	25·9 (23·1 – 28·6)	18·0 (15·4 – 20·5)	-0·3 (-0·4 – -0·2)
Anxiety disorders	20 (19 – 20)	20 (20 – 21)	3379·6 (3169·2 – 3588·8)	5577·1 (5229·9 – 5922·3)	5195·3 (4843·4 – 5534·4)	2·9 (-2·0 – 7·7)	-3·6 (-8·1 – 1·0)	-2·8 (-7·2 – 1·5)

Supplementary Table 3a. Age-standardised rates of death and years of life lost due to premature mortality (YLLs) in 1990 and 2017, both sexes combined, with 95% uncertainty intervals, for Italy and selected western European countries (EU15); countries in alphabetical order

	Age-standardised death rate (per 100 000)				Age-standardised YLL rate (per 100 000)			
	1990		2017		1990		2017	
**Country**	**Rate**	**Rank***	**Rate**	**Rank***	**Rate**	**Rank***	**Rate**	**Rank***
Western Europe**	665 (664 – 665)		412 (403 – 421)		14141 (14118 – 14162)		7901 (7728 – 8082)	
Austria	698 (693 - 704)	26	419 (400 - 439)	19	14778 (14632 - 14931)	20	7924 (7523 - 8354)	18
Belgium	681 (677 - 686)	21	432 (413 - 453)	24	14541 (14411 - 14672)	17	8404 (7980 - 8835)	22
Denmark	729 (723 - 735)	35	462 (439 - 485)	34	15745 (15583 - 15928)	26	8680 (8224 - 9189)	27
Finland	726 (719 - 732)	34	432 (412 - 454)	23	15620 (15465 - 15772)	25	8329 (7904 - 8805)	21
France	622 (620 - 624)	11	373 (357 - 391)	7	13669 (13613 - 13722)	15	7646 (7277 - 8033)	12
Germany	710 (708 - 712)	27	460 (421 - 500)	33	14987 (14936 - 15039)	22	8873 (8081 - 9685)	30
Greece	605 (600 - 609)	8	451 (431 - 471)	31	12663 (12532 - 12800)	6	8778 (8352 - 9233)	28
Ireland	782 (773 - 790)	45	419 (398 - 440)	18	15578 (15379 - 15783)	24	7752 (7351 - 8183)	14
**Italy**	**627 (625 - 629)**	**15**	**368 (352 - 385)**	**5**	**13297 (13235 - 13362)**	**10**	**6709 (6384 - 7038)**	**4**
Luxembourg	720 (710 - 731)	33	431 (402 - 462)	22	15313 (15034 - 15613)	23	7835 (7248 - 8458)	16
Netherlands	637 (632 - 640)	16	436 (417 - 457)	26	13028 (12926 - 13133)	8	7935 (7529 - 8369)	19
Portugal	812 (807 - 817)	57	434 (415 - 453)	25	17817 (17661 - 17980)	38	8232 (7788 - 8656)	20
Spain	624 (622 - 627)	12	370 (355 - 385)	6	13567 (13497 - 13641)	12	6910 (6592 - 7220)	5
Sweden	600 (595 - 605)	6	396 (380 - 412)	13	12146 (12034 - 12263)	3	7146 (6842 - 7470)	8
United Kingdom	697 (695 - 698)	25	450 (446 - 454)	30	14551 (14521 - 14579)	18	8781 (8697 - 8867)	29

* Ranking is on a global scale, considering all 195 countries and territories.
** Western Europe includes additional countries: Andorra, Cyprus, Iceland, Israel, Malta, and Switzerland.

Supplementary Table 3b. Age-standardised rates of years lived with disability (YLDs) and disability-adjusted life-years (DALYs) in 1990 and 2017, both sexes combined, with 95% uncertainty intervals for Italy and selected western European countries (EU15); countries in alphabetical order

	**Age-standardised YLD rate (per 100 000)**				**Age-standardised DALY rate (per 100 000)**			
	**1990**		**2017**		**1990**		**2017**	
**Country**	**Rate**	**Rank***	**Rate**	**Rank***	**Rate**	**Rank***	**Rate**	**Rank***
Western Europe**	10687 (8031 – 13871)		10635 (8012 – 13711)		24828 (22169 – 28000)		18536 (15928 – 21551)	
Austria	10599 (7921 - 13710)	47	10697 (8075 - 13772)	70	25377 (22715 - 28500)	19	18621 (15855 - 21766)	15
Belgium	10861 (8119 - 13976)	66	11061 (8294 - 14363)	98	25402 (22731 - 28483)	20	19465 (16600 - 22709)	27
Denmark	10633 (7985 - 13807)	52	10751 (8070 - 14054)	76	26379 (23756 - 29533)	24	19431 (16658 - 22632)	26
Finland	11316 (8502 - 14633)	92	11193 (8421 - 14429)	108	26936 (24075 - 30247)	29	19522 (16680 - 22827)	28
France	10234 (7673 - 13315)	30	10187 (7620 - 13141)	43	23903 (21323 - 26984)	12	17833 (15283 - 20836)	9
Germany	10764 (8043 - 14016)	58	10707 (8116 - 13881)	72	25751 (23045 - 28998)	22	19580 (16739 - 22901)	29
Greece	10600 (7952 - 13668)	49	10618 (7995 - 13643)	63	23263 (20603 - 26267)	5	19397 (16746 - 22519)	25
Ireland	10839 (8106 - 13919)	62	10871 (8115 - 13999)	83	26417 (23647 - 29514)	25	18623 (15963 - 21765)	16
**Italy**	**10609 (7920 - 13644)**	**50**	**10313 (7684 - 13277)**	**48**	**23906 (21233 - 26958)**	**13**	**17022 (14382 - 20025)**	**4**
Luxembourg	11488 (8616 - 14803)	102	11301 (8507 - 14509)	118	26801 (23936 - 30105)	27	19136 (16256 - 22474)	22
Netherlands	10818 (8092 - 13932)	61	10781 (8110 - 13886)	77	23846 (21137 - 26989)	10	18716 (15949 - 21845)	19
Portugal	11143 (8385 - 14470)	80	10704 (8077 - 13923)	71	28960 (26193 - 32223)	40	18936 (16169 - 22175)	20
Spain	10281 (7727 - 13303)	34	10093 (7542 - 13079)	36	23848 (21298 - 26871)	11	17003 (14459 - 19960)	3
Sweden	10779 (8080 - 13972)	59	10857 (8126 - 14082)	82	22925 (20220 - 26108)	3	18002 (15163 - 21162)	11
United Kingdom	11071 (8328 - 14297)	77	11340 (8549 - 14652)	121	25623 (22890 - 28847)	21	20121 (17338 - 23416)	32

* Ranking is on a global scale, considering all 195 countries and territories.
** Western Europe includes additional countries: Andorra, Cyprus, Iceland, Israel, Malta, and Switzerland.
